# Synaptic reshaping of plastic neuronal networks by periodic multichannel stimulation with single-pulse and burst stimuli

**DOI:** 10.1371/journal.pcbi.1010568

**Published:** 2022-11-03

**Authors:** Justus A. Kromer, Peter A. Tass

**Affiliations:** Department of Neurosurgery, Stanford University, Stanford, California, United States of America; Université Paris Descartes, Centre National de la Recherche Scientifique, FRANCE

## Abstract

Synaptic dysfunction is associated with several brain disorders, including Alzheimer’s disease, Parkinson’s disease (PD) and obsessive compulsive disorder (OCD). Utilizing synaptic plasticity, brain stimulation is capable of reshaping synaptic connectivity. This may pave the way for novel therapies that specifically counteract pathological synaptic connectivity. For instance, in PD, novel multichannel coordinated reset stimulation (CRS) was designed to counteract neuronal synchrony and down-regulate pathological synaptic connectivity. CRS was shown to entail long-lasting therapeutic aftereffects in PD patients and related animal models. This is in marked contrast to conventional deep brain stimulation (DBS) therapy, where PD symptoms return shortly after stimulation ceases.

In the present paper, we study synaptic reshaping by periodic multichannel stimulation (PMCS) in networks of leaky integrate-and-fire (LIF) neurons with spike-timing-dependent plasticity (STDP). During PMCS, phase-shifted periodic stimulus trains are delivered to segregated neuronal subpopulations. Harnessing STDP, PMCS leads to changes of the synaptic network structure. We found that the PMCS-induced changes of the network structure depend on both the phase lags between stimuli and the shape of individual stimuli. Single-pulse stimuli and burst stimuli with low intraburst frequency down-regulate synapses between neurons receiving stimuli simultaneously. In contrast, burst stimuli with high intraburst frequency up-regulate these synapses. We derive theoretical approximations of the stimulation-induced network structure. This enables us to formulate stimulation strategies for inducing a variety of network structures. Our results provide testable hypotheses for future pre-clinical and clinical studies and suggest that periodic multichannel stimulation may be suitable for reshaping plastic neuronal networks to counteract pathological synaptic connectivity. Furthermore, we provide novel insight on how the stimulus type may affect the long-lasting outcome of conventional DBS. This may strongly impact parameter adjustment procedures for clinical DBS, which, so far, primarily focused on acute effects of stimulation.

## Author summary

Synaptic dysfunction accompanies several brain disorders, such as Alzheimer’s, Parkinson’s disease and obsessive compulsive disorder. For therapeutic purposes, stimulation is delivered to disease-related brain areas. Brain stimulation therapies that manipulate synaptic connections in disease-related brain areas may provide long-lasting symptom relief.

In this computational and theoretical study, we study periodic multichannel stimulation, a stimulation technique that allows for manipulating selected synaptic populations. Stimulus trains are delivered to multiple neuronal subpopulations in order to trigger neuronal responses. Using a model network of leaky integrate-and-fire neurons with spike-timing-dependent plasticity and theoretical analysis, we show how the relative timings between and the shape of delivered stimuli can be tuned to down-regulate certain synaptic connections while up-regulating others. Single-pulse stimuli triggered precise neuronal responses and were suitable for inducing a variety of synaptic network structures. When burst stimuli were employed, tuning the intraburst frequency allowed for distinguishing between down- and up-regulation of synaptic connections within individual neuronal subpopulations. Our work provides a theoretical basis for selecting suitable stimulation parameters for inducing long-lasting therapeutic effects in patients suffering from neurological disorders.

## Introduction

Several brain disorders are accompanied by synaptic dysfunction and/or pathological synaptic connectivity [1]. Such disorders include Alzheimer’s disease [2], attention-deficit/hyperactivity disorder [3, 4], obsessive compulsive disorder (OCD) [5, 6] and Parkinson’s disease (PD) [7, 8]. Pathological connectivity may arise from different biological mechanisms that may, for instance, lead to the loss of synapses in disease-specific brain areas and/or to an impairment of synaptic plasticity [1, 7, 9].

Brain stimulation may induce long-lasting changes of synaptic connectivity by utilizing synaptic plasticity. Corresponding direct and indirect evidence has been provided by several experimental studies [10–16]. For instance, in rat brain slices, high-frequency stimulation of the subthalamic nucleus (STN), a common target area for deep brain stimulation (DBS) in PD, induced changes of synaptic weights that outlasted stimulation for at least about half an hour (the maximum observation window considered in the experiments) [10, 14]. Another study showed that synaptic weight changes are strongly affected by the applied pattern of electrical stimuli and the concentration of neuromodulators, such as dopamine [11]. Indirect evidence of stimulation-induced synaptic plasticity has been provided by experiments on transcranial magnetic stimulation of the visual cortex [12, 13]. There, aftereffects remained stable for hours after cessation of stimulation. Counteracting pathological synaptic connectivity using brain stimulation may improve current treatments and enable to develop novel treatments that aim at long-lasting symptom relief for patients suffering from neurological disorders.

One stimulation technique that was found to induce long-lasting symptom relief in PD is coordinated reset stimulation (CRS). CRS was computationally developed to specifically induce long-lasting desynchronization effects [17, 18]. It is a multisite stimulation technique during which spatio-temporal stimulus patterns are delivered to multiple neuronal subpopulations [17, 19]. CRS was tested in the 1-methyl-4-phenyl-1,2,3,6-tetrahydropyridine (MPTP)-monkey model of PD using downscaled DBS electrodes [20]. Two hours of CRS delivered to the STN on five consecutive days led to a significant reduction of akinesia, outlasting stimulation by several weeks [20], see also [21, 22]. In PD patients, CRS unilaterally administered to the STN led to significant bilateral motor improvement, accumulating over consecutive days [23]. In contrast, symptoms return shortly after cessation of high-frequency DBS, the current standard of care for medically refractory PD [20, 24]. Recently, CRS was also delivered using non-invasive vibrotactile fingertip stimulation and clinically significant long-lasting therapeutic effects were observed [25, 26]. Currently, it is hypothesized that the long-lasting therapeutic effects of CRS result from stimulation-induced synaptic reshaping in related brain areas, driving the neuronal network into more favorable attractors, in this way restoring physiological function after cessation of stimulation [18].

In order to understand synaptic reshaping of plastic neuronal networks, theoretical and computational papers studied neuronal networks with spike-timing-plasticity (STDP) [27–33]. Synaptic reshaping due to STDP depends on the time lags between postsynaptic and presynaptic spikes [34, 35], or higher-order statistics of the spike times [31, 36]. The type of plasticity determines the interplay of neuronal activity and network connectivity [32, 33, 37, 38]. In many brain areas, STDP strengthens synapses if the postsynaptic neuron repetitively spikes shortly after the presynaptic one and weakens synapses if the postsynaptic neuron spikes shortly before the presynaptic neuron [35]. STDP may stabilize certain neuronal activity patterns [39, 40], and it may lead to the formation of several network motifs [32, 41–45], including neuronal assemblies. The latter are believed to play a key rule in memory formation [46]. STDP may also lead to the coexistence of different stable states, such as synchronized, desynchronized, and cluster states [18, 38, 47–51].

Of particular interest is the response of plastic neuronal networks to external stimulation. Theoretical and computational studies investigated synaptic reshaping in response to periodic input [52, 53]. Other studies found that collective spiking events, for instance, in response to a stimulus, strongly impact synaptic reshaping. These events led to qualitatively different synaptic reshaping in neuronal networks with dendritic delays [28] and neuronal networks with axonal delays [29]. Such reshaping was also analyzed in networks with both axonal and dendritic delays [30]. In networks with both types of delays, stimulation resulted in the formation of distinct network motifs [44]. Another series of computational studies focused on the capability of stimulation to induce long-lasting desynchronization by down-regulating pathological connectivity (decoupling). This decoupling of neuronal subpopulations may drive the network into the attractor of a stable desynchronized state and lead to long-lasting desynchronization effects that outlast stimulation [18, 54–59].

In order to identify stimulation patterns that may induce favorable changes of synaptic connectivity, a recent theoretical study presented an approximation of the mean rate of synaptic weight change during ongoing delivery of spatio-temporal stimulus patterns [60]. The results were derived in the limit of stimulation-controlled spiking activity, where neuronal spiking follows the stimulus pattern [60]. In that limit, synaptic reshaping is controlled by two main aspects of the stimulation pattern: the spatio-temporal correlations between stimulus deliveries and the statistics of neuronal spiking responses to individual stimuli [60]. The former aspect is controlled by the delivered stimulus pattern as characterized by the timings and locations of individual stimulus deliveries. Possible patterns include periodic stimulation, CRS, or randomized stimulus patterns. Previous studies have investigated synaptic reshaping for several stimulus patterns by assuming sharp distributions of stimulus-triggered neuronal spikes [60–64]. Less is known about how the statistics of stimulus-triggered spikes impact synaptic reshaping. These statistics are strongly affected by the stimulus type. In previous computational and theoretical studies, stimulation was delivered using charge-balanced electrical single-pulse stimuli that resembled direct electrical stimulation of the neurons’ somata [60–63]. Other computational studies analyzed synaptic reshaping during stimulation employing electrical burst stimuli [18, 54–56, 58], sensory stimuli, which resembled stimulation of neuronal fibers [56, 65], and vibrotactile stimuli, modeling neuronal responses to vibratory stimulation of the fingertips [26]. It is currently unknown how such complex stimuli affect the stimulation-induced network structure and whether certain stimulus types are more favorable to induce synaptic reshaping than others.

**Fig 1:**
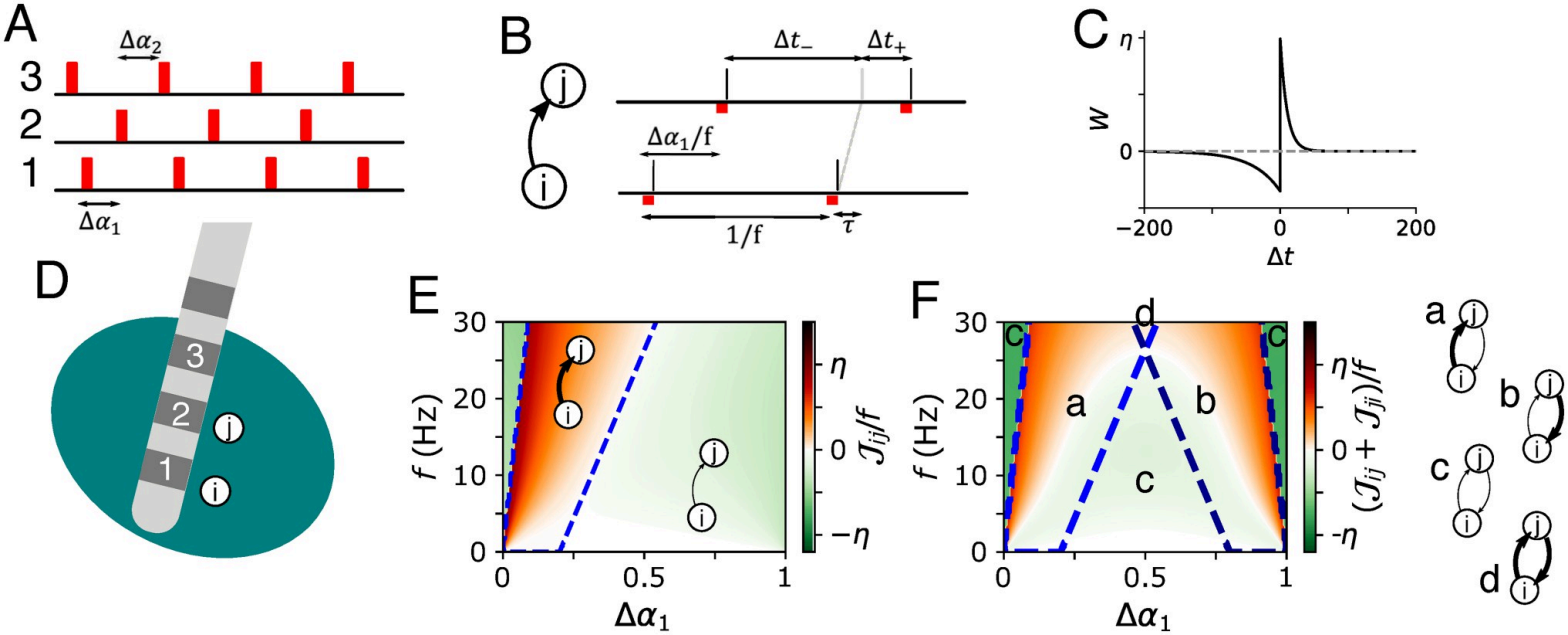
Reshaping of synaptic connectivity by PMCS. A: During PMCS, periodic stimulus trains (red) are delivered to multiple neuronal subpopulations (1,2,3) with phase shifts (Δ*α*_1_,Δ*α*_2_). D: Schematic of PMCS delivery using a multisite DBS electrode where phase-shifted stimulus trains are delivered to three contacts (1,2,3). Neurons are mostly affected by stimulus trains that are delivered to nearby stimulation contacts, neuron *i* by stimuli delivered to contact 1 and neuron *j* by stimuli delivered to contact 2. B: For illustration, in this figure we assume that neurons respond with a single spike (black vertical bar) of high fidelity to a stimulus (red). During stimulation, the spike train of the postsynaptic neuron, *j*, and that of the presynaptic neuron, *i*, become time shifted by Δ*α*_1_/*f*, where *f* is the stimulation frequency. Due to STDP, time lags, Δ*t*, between postsynaptic spikes and delayed presynaptic spike arrivals (gray) lead to weight updates, *W*, according to the STDP function (C, see Eq 10). Negative weight updates result from pairings of presynaptic spike arrivals with earlier postsynaptic spikes (Δ*t*_−_ < 0) and positive updates from pairings with later postsynaptic spikes (Δ*t*_+_ > 0). E: The sign of the overall weight update per inter-stimulus interval (ISI), $J_{ij}/f\approx W\left(\Delta t_{-}\right)+W\left(\Delta t_{+}\right)$, determines whether the synapse between neurons *i* and *j* is up-regulated (thick arrow) or down-regulated (thin arrow). F: If the neurons are bidirectionally coupled, PMCS may either induce effective unidirectional coupling (a,b), bidrectional coupling (d), or lead to decoupling (c). Blue dashed lines in panels E and F separate parameter regions with $J_{ij}>0$ from regions with $J_{ij}<0$. Dark blue dashed lines separate corresponding regions for $J_{ji}$.

In the present paper, we deliver *periodic multichannel stimulation* (PMCS) with different types of stimuli to induce a desired network structure in neuronal networks with STDP. During PMCS, phase-shifted periodic stimulus trains are delivered to multiple neuronal subpopulations. Depending on the phase shifts between stimulus trains delivered to synaptically interconnected subpopulations, postsynaptic neurons tend to spike either before or after presynaptic ones. Due to STDP, stimulation may then either induce bidirectional coupling, unidirectional coupling, or lead to a decoupling of the neuronal subpopulations. This basic mechanism of PMCS is illustrated in Fig 1. The PMCS-induced synaptic network structure is determined by the phase shifts between the stimulus trains. PMCS can be understood as a generalization of classic CRS, with fixed stimulus sequence [17, 19], as it allows for arbitrary phase shifts between stimulus trains.

We provide a detailed computational and theoretical analysis of PMCS, which led to promising stimulation strategies for possible clinical applications. We derive a theoretical basis for studying synaptic reshaping for a broad class of stimuli that may trigger complex neuronal spiking responses, such as bursts. Our theoretical predictions are compared to numerical simulations of PMCS of networks of leaky integrate-and-fire (LIF) neurons with STDP using electrical single-pulse and burst stimuli. We found that the same PMCS pattern administered using different types of stimuli leads to qualitatively different network structures. This suggests that the selection of the stimulus shape may not only impact the recruitment of neuronal subpopulations but also influence how stimulation affects plastic synaptic connections. Furthermore, we show how phase shifts between stimulus trains can be tuned to down-regulate synaptic connections between certain subpopulations while up-regulating others. Our results provide testable hypotheses for possible pre-clinical and clinical studies. For instance, PMCS could be delivered to down-regulate pathological synaptic connections in order to induce long-lasting therapeutic effects in patients suffering from neurological disorders, e.g., PD [23, 26].

The present paper is organized as follows. First, we present our analysis of the statistics of stimulus-triggered spikes for single-pulse and burst stimuli in networks of LIF neurons. Then, we provide a systematic theoretical and computational analysis of synaptic reshaping during PMCS. Based on this analysis, we present stimulation strategies for inducing a variety of network structures and test them in the LIF network model. Then, we discuss our results and their potential implications for DBS. Finally, we present the details of the LIF network model and methods used throughout the paper as well as the derivation of our theoretical approximations.

## Results

We studied the response of networks of excitatory LIF neurons with STDP to PMCS. The dynamics of the LIF neurons’ membrane potentials was adjusted to that of tonically spiking neurons in the rat STN [66]. The networks were prepared in a stationary state with synchronized spiking and strong synaptic connections with mean synaptic weight 〈*w*〉 ≈ 0.38 (Methods). This resembled a pathological state in which strong synaptic connectivity supports excessive neuronal synchrony, e.g., during PD. Then, stimulation was delivered using either charge-balanced single-pulse stimuli, referred to as single-pulse stimuli in the following, or bursts of charge-balanced pulses, referred to as burst stimuli in the following (see Methods for details).

### Statistics of stimulus-triggered spikes depends on stimulus shape

First, we studied the statistics of stimulus-triggered spikes. To this end, we administered periodic stimulus trains to a portion of the LIF neurons using either single-pulse or burst stimuli.

**Fig 2:**
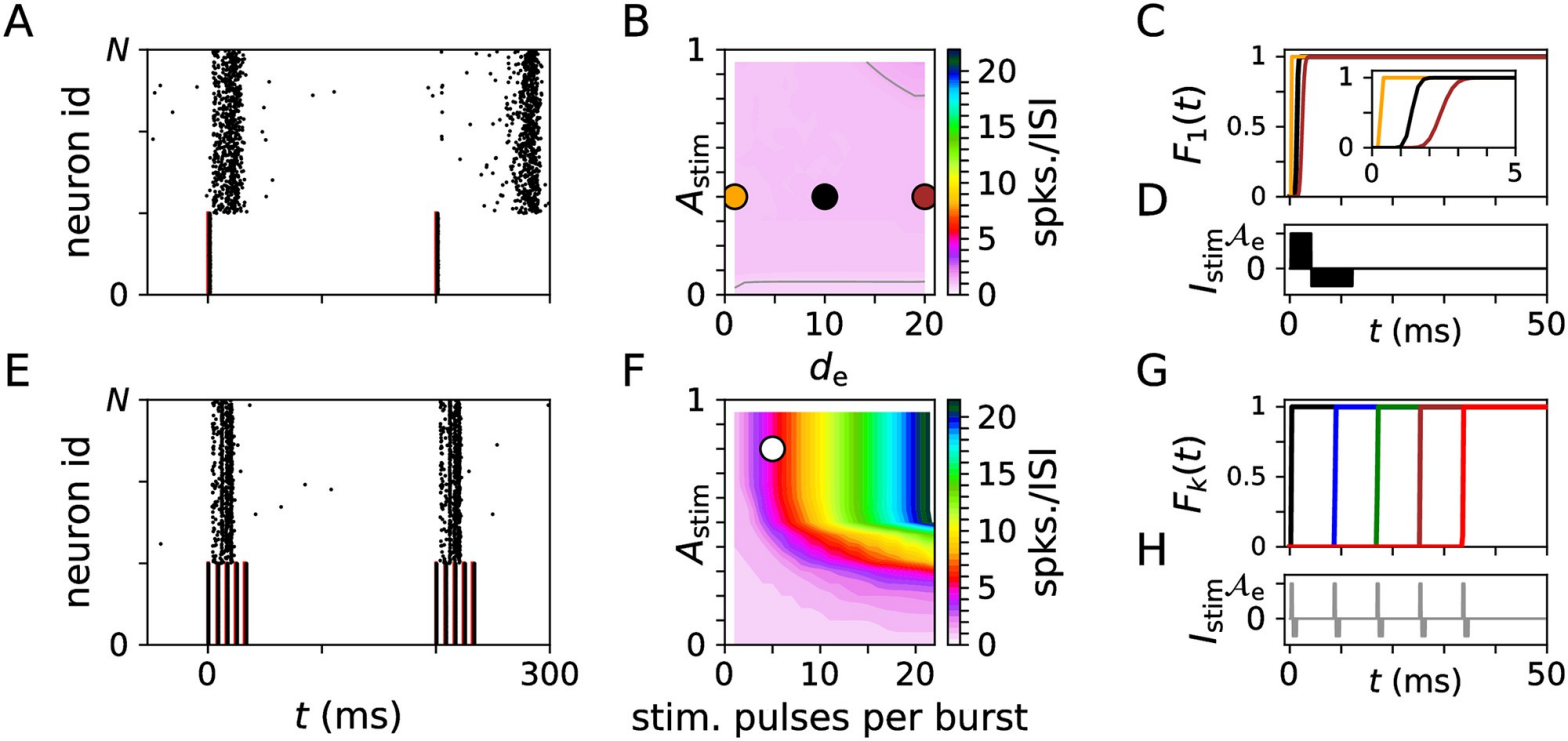
Statistics of LIF neurons’ spiking responses to periodic delivery of single-pulse (top) and burst stimuli (bottom). A: Raster plot of spiking activity of a network of 10^3^ LIF neurons during periodic single-pulse stimulation (red bars) of 333 neurons. B: Mean number of spikes per ISI and neuron as function of the dimensionless stimulation amplitude, *A*_stim_, and pulse width, *d*_e_ (Methods). Gray contour lines correspond to 0.75 and 1.25 spikes per ISI and neuron (from bottom to top). C: Cumulative distribution function of the timing of the first spike of LIF neurons after stimulus delivery $F_{1}\left(t\right)=\int_{0}^{t}du\;\Lambda_{1}\left(u\right)$ (Methods) for the stimulus parameters marked orange, black, and brown, respectively, in panel B. The inset zooms into the first 5 ms. The stimulation current, *I*_stim_, corresponding to *d*_e_ = 10 is shown in panel D. E: Same as A but for burst stimuli with five pulses per burst and an intraburst frequency of 120 Hz. F: Mean number of spikes per ISI and neuron as function of the number of stimulus pulses per burst and the stimulation amplitude. G: Cumulative distribution function of the timing of the *k*th spike per ISI $F_{k}\left(t\right)=\int_{0}^{t}du\;\Lambda_{k}\left(u\right)$ for the parameter set marked white in panel F. Color code: *F*_1_(*t*) (black), *F*_2_(*t*) (blue), *F*_3_(*t*) (green), *F*_4_(*t*) (brown), and *F*_5_(*t*) (red). H: Corresponding stimulation current, *I*_stim_. Parameters: *f* = 5 Hz (all); *A*_stim_ = 0.4, and *d*_e_ = 10 (A,C,D) and *d*_e_ = 1 (C, orange) and *d*_e_ = 20 (C, brown); and *A*_stim_ = 0.8, *d*_e_ = 1, and 120 Hz intraburst frequency (E, F, G, H).

Results are shown in Fig 2. Spiking of stimulated neurons entrained with stimuli (Fig 2A and 2E). Stimulus-triggered spiking responses depended on the shape of administered stimuli (Fig 2C, 2D, 2G and 2H). We measured the mean number of spikes per ISI (Fig 2B and 2F). Periodic single-pulse stimulation led to at most two spikes per ISI (Fig 2B). The first spike occurred shortly after stimulus onset. For sufficiently strong and broad stimuli, a second spike occurred towards the end of the ISI, when the LIF neurons’ refractory periods had past and neurons became susceptible to excitatory synaptic input from the rest of the network. In contrast, strong burst stimuli triggered several spikes per ISI, typically one spike per pulse (Fig 2F).

For statistical analysis, we evaluated the distribution of the *k*th spike time during an ISI, Λ_*k*_(*t*), (Methods) and the corresponding cumulative distribution function $F_{k}\left(t\right)=\int_{0}^{t}du\;\Lambda_{k}\left(u\right)$. The shape of *F*_*k*_(*t*) for single-pulse stimuli depended on the pulse duration, *d*_*e*_. Short pulses typically resulted in step-like *F*_*k*_(*t*) whereas long pulses led to broad Λ_*k*_(*t*) resulting in a slower increase of *F*_*k*_(*t*), mostly during the excitatory part of the stimulus pulse (Fig 2C). For burst stimuli, individual stimulus pulses led to step-like increases of *F*_*k*_(*t*) (Fig 2G and Methods), indicating that spiking occurred shortly after the onset of the *k*th stimulation pulse (Fig 2G).

Next, we studied how PMCS delivered to *M* neuronal subpopulations affects the strength of synaptic connections. For illustration, we restricted our analysis to *M* ≤ 3. The spatio-temporal characteristics of the PMCS pattern are controlled by the phase lags Δ*α*_*k*_, *k* = 1, 2, ‥, *M* − 1, between stimulus deliveries to adjacent neuronal subpopulations (Fig 1A). Note that simulations were performed for networks of LIF neurons with homogeneous synaptic connectivity. Thus, spatial adjacency was solely induced by the grouping of neurons into subpopulations according to their indices. Neurons in the same subpopulation received stimuli simultaneously.

### Synaptic weight dynamics during PMCS delivered to two subpopulations

**Fig 3:**
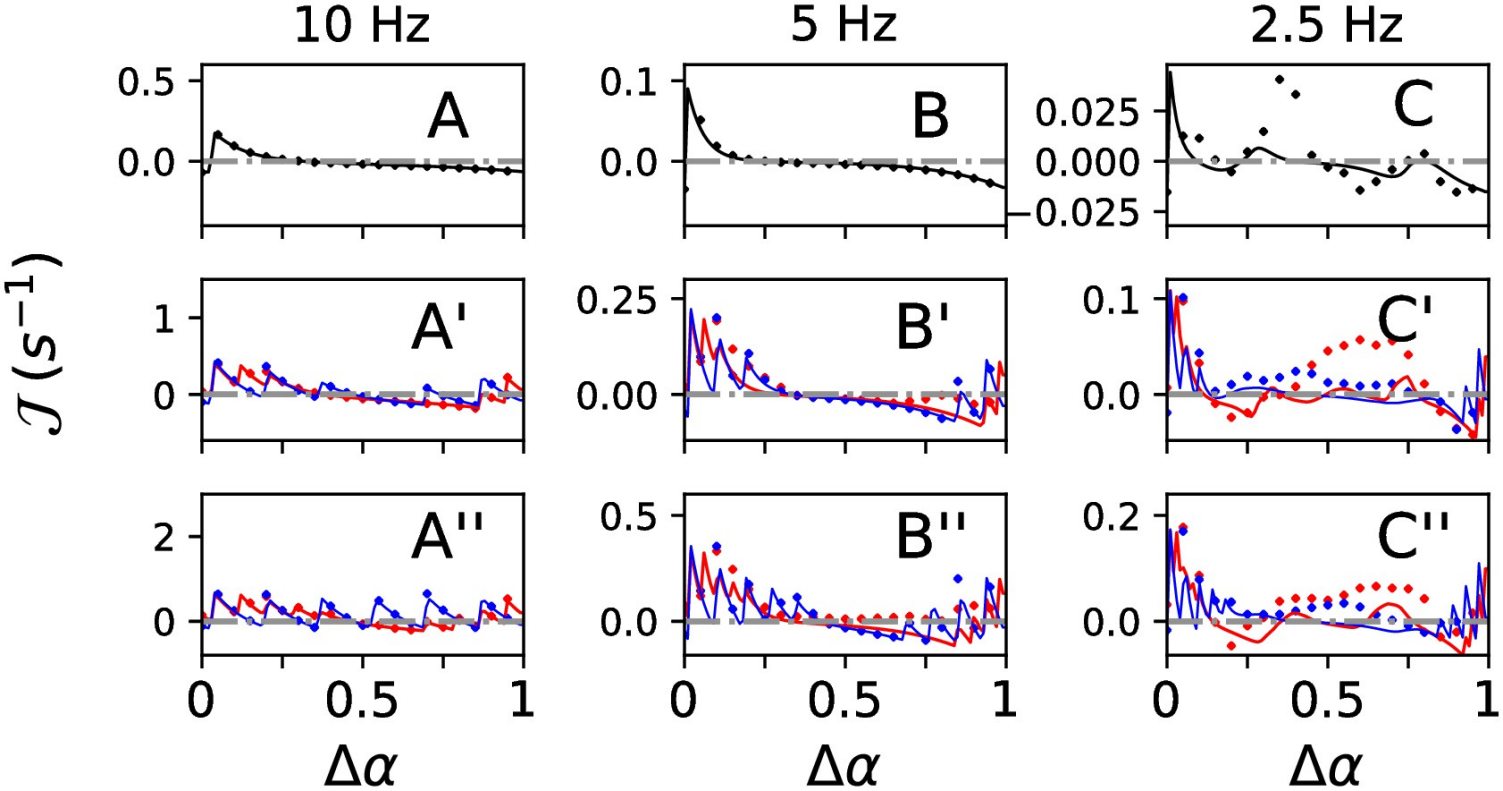
Effect of PMCS with (*M* = 2) on the mean weight of synapses with postsynaptic neuron in subpopulation 2 and presynaptic neuron in subpopulation 1 as function of the phase lag, Δ*α*, between stimulus trains delivered to the two subpopulations. Panels show results for single-pulse (A-C) and burst stimuli with three (A’-C’) and five (A”-C”) pulses per burst. Results for high (120 Hz) and low (60 Hz) intraburst frequencies are shown in red and blue, respectively. Curves show theoretical approximations $\langle J^{\infty }\rangle$ (Eq 19) and markers $J^{\text{est}}$ obtained from simulations and averaged over five network realizations and initial conditions (Methods). Parameters: *d*_e_ = 1 and *A*_stim_ = 0.4 (A,B,C), *A*_stim_ = 0.8 (A’-C’, A”-C”). Note that different intervals are plotted on the y-axes.

We considered the case of *M* = 2 equally sized subpopulations and delivered PMCS with phase lag Δ*α* ≔ Δ*α*_1_ and stimulation frequency *f* to the network of excitatory LIF neurons. We recorded the trace of the mean weight of synapses between neurons in subpopulation one and neurons in subpopulation two, 〈*w*_1→2_(*t*)〉, and estimated the mean rate of weight change, $J^{\text{est}}$ (Methods). Simulation results for different types of stimuli and stimulation frequencies are shown in Fig 3.

In addition, we derived a theoretical estimate for the mean rate of synaptic weight change, $\langle J^{\infty }\left(\Delta \alpha ,f,\xi \right)\rangle$, using the distributions of stimulus-triggered spikes, Λ_*k*_(*t*). $\langle J^{\infty }\left(\Delta \alpha ,f,\xi \right)\rangle$ is given in Eq 19 in the Methods section. Δ*α* is the phase lag between periodic stimulus trains delivered to the postsynaptic and presynaptic subpopulation and is given by the time shift between stimuli divided by the stimulation period (Fig 1B). *ξ* is the difference between axonal and dendritic delays. During the derivation of $\langle J^{\infty }\left(\Delta \alpha ,f,\xi \right)\rangle$, we assumed that Λ_*k*_(*t*) approximated the spiking of neurons in individual subpopulations well, even if spiking of other subpopulations was altered by stimulation.

In Fig 3, we compare $\langle J^{\infty }\left(\Delta \alpha ,f,\xi \right)\rangle$ with $J^{\text{est}}$. For single-pulse stimuli, synaptic weight changes mainly resulted for small positive and negative phase lags, Δ*α* (modulo 1) (Fig 3A and 3B). For slow single-pulse stimulation, larger phase lags also contributed to weight changes (Fig 3C). For burst stimuli, weight changes occurred for a wide range of phase lags (Fig 3A’, 3B’, 3C’, 3A”, 3B” and 3C”). Faster stimulation and increasing the number of pulses per stimulus resulted in larger positive and negative weight updates.

Overall, $\langle J^{\infty }\left(\Delta \alpha ,f,\xi \right)\rangle$ approximated the shape of $J^{\text{est}}$ well for fast stimulation (Fig 3A, 3A’, and 3A”).

### Theoretical approximation of PMCS-induced weight dynamics

Next, we considered PMCS with an arbitrary number of subpopulations *M*. Using $\langle J^{\infty }\left(\Delta \alpha ,f,\xi \right)\rangle$, we approximated the mean weight 〈*w*_*x*→*y*_(*t*)〉 of synapses interconnecting the subpopulations *x* and *y* at time *t* after stimulation onset. Here and in the following, the indices *x* and *y* refer to the presynaptic and postsynaptic neuronal subpopulations, and the indices *i* and *j* to the presynaptic and postsynaptic neuron, respectively. Given 〈*w*_*x*→*y*_(*t*)〉 at stimulation onset *t* = *t*_0_, 〈*w*_*x*→*y*_(*t*)〉 at time *t* during stimulation (*t* > *t*_0_) is approximately given by

$$\begin{array}{c} \left\langle w_{x\to y}\left(t\right)\right\rangle \approx [\left\langle w_{x\to y}\left(t_{0}\right)\right\rangle +S\left(\left\langle J^{\infty }\left(\Delta \phi_{xy},f,\xi \right)\right\rangle ,\left\langle w_{x\to y}\left(t_{0}\right)\right\rangle \right)\left(t-t_{0}\right){]}_{\text{clip},[0,1]} \end{array}$$ (1)

Here, [*x*]_clip,[0,1]_ = *x* for *x* ∈ [0, 1], [*x*]_clip,[0,1]_ = 0 for *x* < 0, and [*x*]_clip,[0,1]_ = 1 for *x* > 1 accounts for the hard bounds for individual synaptic weights (Methods). The function

$$\begin{array}{c} S\left(J,w\right)≔\{\begin{array}{cc} wJ, & J\leq 0\\ (1-w)J, & J>0 \end{array} \end{array}$$ (2)

describes a linear decay/increase of 〈*w*_*x*→*y*_(*t*)〉 during stimulation with the mean rate of weight change J. Δ*ϕ*_*xy*_ is the phase shift between stimulus deliveries to the postsynaptic subpopulation *y* and the presynaptic subpopulation *x* and follows from the phase lags Δ*α*_*k*_ characterizing the PMCS pattern (Fig 1A). Eq 1 was derived based on the assumption that the weights of individual synapses are close to the hard bounds prior to stimulation onset, i.e, either *w*_*i*→*j*_ ≈ 0 or *w*_*i*→*j*_ ≈ 1 at time *t*_0_. This is typically observed in networks with additive STDP and hard bounds, Ref. [37, 67] and was observed in a previous study using a similar LIF model [60]. Eq 1 either accounts for a linear increase of the weights of weak synapses (J>0) or a linear decrease of the weights of strong synapses (J<0).

For a PMCS pattern with *M* separately stimulated neuronal subpopulations, the *M*^2^ phase lags Δ*ϕ*_*xy*_ can be expressed through the *M* − 1 phase lags between stimulus trains delivered to subpopulations with adjacent indices Δ*α*_*k*_ (Fig 1A) by

$$\begin{array}{c} \Delta \phi_{xy}=\{\begin{array}{cc} (\sum_{k=x}^{y-1}\Delta \alpha_{k})\;\text{mod}\;1, & y>x\\ 0, & y=x\\ (-\sum_{k=y}^{x-1}\Delta \alpha_{k})\;\text{mod}\;1, & y<x \end{array}. \end{array}$$ (3)

Using the phase lags Δ*ϕ*_*xy*_ and $\langle J^{\infty }\left(\Delta \alpha ,f,\xi \right)\rangle$ in Eq 1, we obtained an approximation for the time-dependent mean synaptic weight 〈*w*(*t*)〉 during stimulation

$$\begin{array}{c} \left\langle w\left(t\right)\right\rangle \overset{\text{hom}.}{\approx }\frac{1}{M^{2}}\sum_{x,y=1}^{M}\left\langle w_{x\to y}\left(t\right)\right\rangle . \end{array}$$ (4)

Here, we assumed a homogeneous network with *M* equally sized subpopulations, as realized in our LIF network model. Note that while the mean synaptic weights of synapses interconnecting the individual subpopulations, 〈*w*_*x*→*y*_(*t*)〉, evolve approximately linear in time until they reach one of the hard bounds (Eq 1), the overall mean synaptic weight, 〈*w*(*t*)〉, may possess a more complex dynamics (Eq 4).

### PMCS induces changes of synaptic connectivity

**Fig 4:**
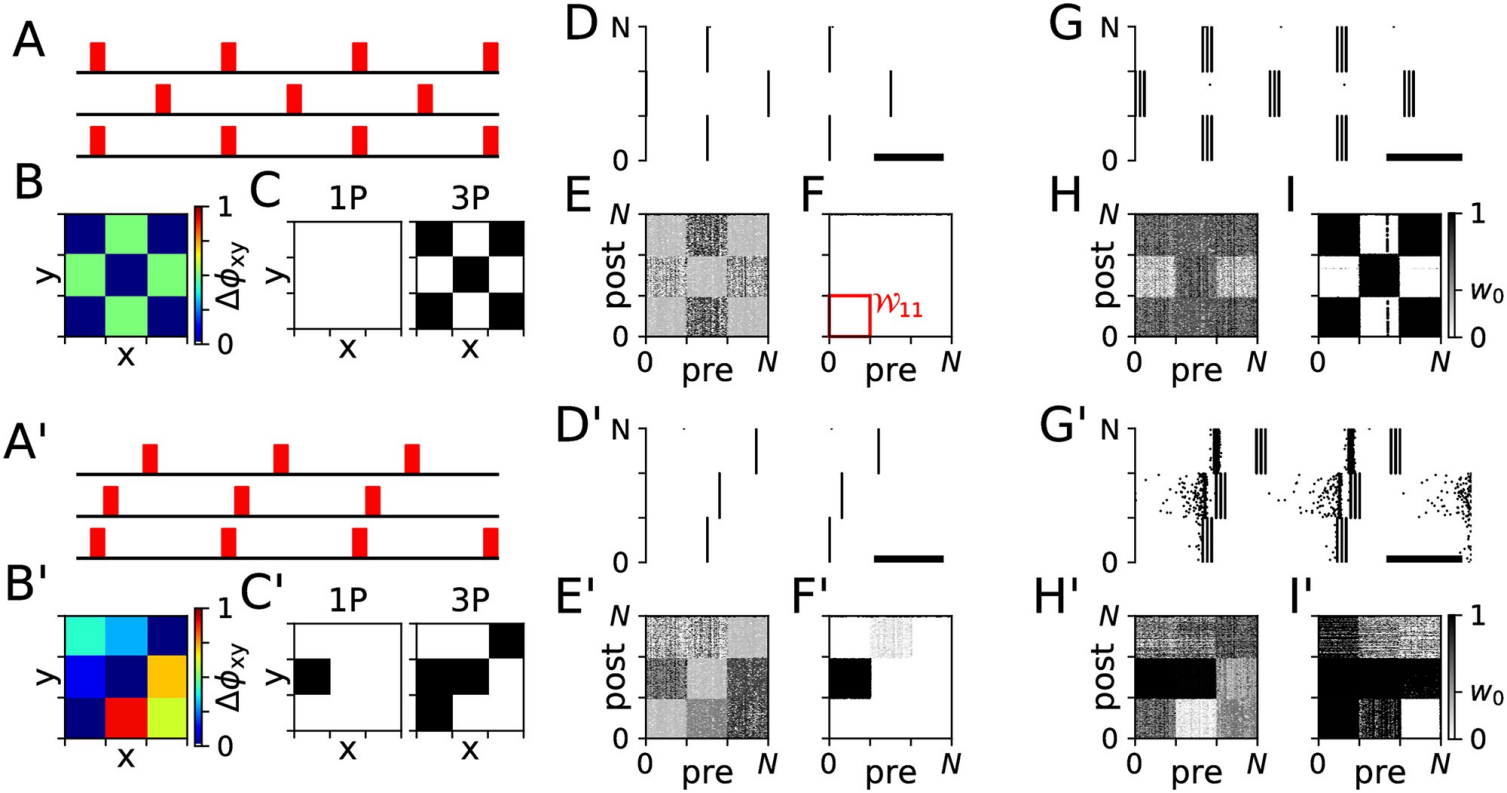
PMCS-induced network structure depends on phase lags and stimulus type. Panels A-I show results for the PMCS pattern illustrated in panel A. Red rectangles mark stimuli. Individual stimuli were either charge-balanced single-pulse (D-F) or burst stimuli (G-I). B: Phase shifts, Δ*ϕ*_xy_, between stimulus trains delivered to the postsynaptic subpopulation *y* and presynaptic subpopulation *x*. C: lim_*t*→∞_〈*w*_*x* → *y*_(*t*)〉 obtained from Eq 1 for single-pulse stimuli (1P) and burst stimuli with three pulses (3P) (see panel I for color code). Panels D-F show a raster plot of simulated spiking activity of the LIF network model 1000 sec after stimulation onset (D), and snapshots of the network’s synaptic weight matrix W taken 20 sec (E) and 1000 sec (F) after stimulation onset. After 1000 sec, acute effects of stimulation have fully developed. The scale bar refers to a 100 ms time interval. The block $W_{11}$ of the synaptic weight matrix is marked red in panel F. G-I: Same as D-F but for burst stimuli with three pulses per burst and an intraburst frequency of 120 Hz. A’-I’: Same as A-I but for the PMCS pattern illustrated in panel A’. Parameters: *f* = 5 Hz; *A*_stim_ = 0.4 (single-pulse stimuli) and 0.8 (burst stimuli), *d*_e_ = 1. Δ*α*_1/2_ = 0.5 (A-I), and Δ*α*_1_ = 0.1 and Δ*α*_2_ = 0.3 (A’-I’).

We simulated the LIF network during stimulation with different PMCS patterns and studied the stimulation-induced network structure. Simulation results for two PMCS patterns with *M* = 3 subpopulations are presented in Fig 4. The two patterns are illustrated in Fig 4A and 4A’, respectively. Respective results are presented in Fig 4A–4I and 4A’–4I’. For *M* = 3 the relative timing of stimulus trains can be characterized by the two phase lags Δ*α*_1_ and Δ*α*_2_ (Fig 1A). Results for the two PMCS patterns and single-pulse stimuli are shown in Fig 4D, 4E, 4F, 4D’, 4E’ and 4F’, respectively. Results for burst stimuli are shown in Fig 4G–4I and 4G’-4I’, respectively. The delivered PMCS pattern strongly affected the stimulation-induced network structure, which we visualize using the synaptic weight matrix W after 1000 seconds of PMCS (Fig 4F, 4F’, 4I and 4I’, respectively).

For a more detailed analysis of the stimulation-induced network structure, we studied the block structure of W. Following, $W_{xy}$ refers to the block containing weights of synapses between the presynaptic neuronal subpopulation *x* and the postsynaptic subpopulation *y* (see Fig 4F). Depending on the phase lags Δ*α*_1_ and Δ*α*_2_, PMCS either up-regulated or down-regulated synapses between individual neuronal subpopulations. This resulted either in high or low weights in the corresponding blocks $W_{xy}$. Theoretical estimates of lim_*t*→∞_〈*w*_*x*→*y*_(*t*)〉 (Eq 1) are presented in Fig 4C and 4C’ for the two PMCS patterns, respectively.

**Fig 5:**
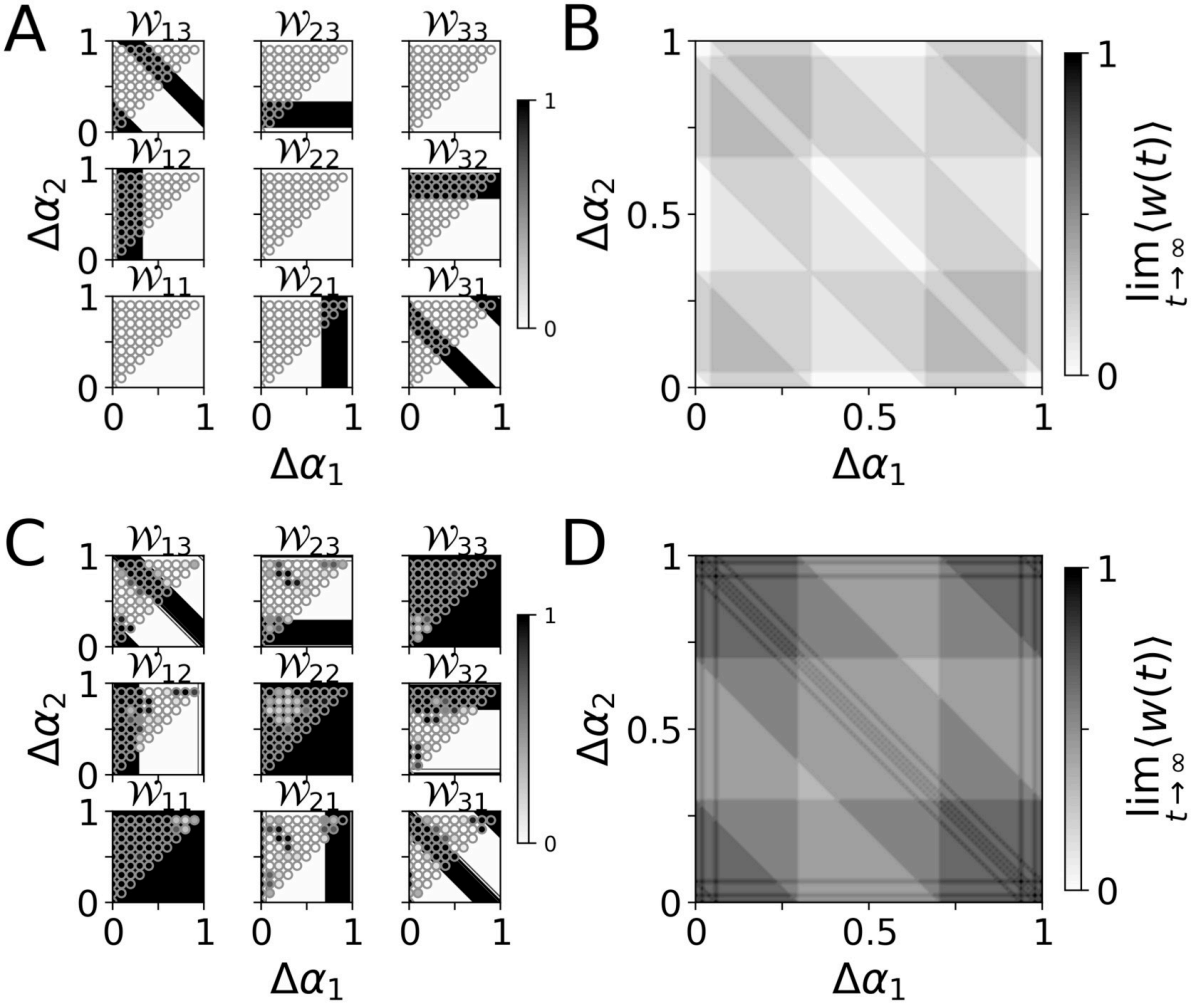
PMCS-induced network structure depends on phase lags between stimulus trains. Predicted block structure of the synaptic weight matrix W after long PMCS employing single-pulse stimuli (A, B) and PMCS employing burst stimuli consisting of three pulses (C, D). A: lim_*x*→*y*_〈*w*_*x*→*y*_(*t*)〉, obtained from Eq 1, (color map) is compared to simulation results for the LIF network model after 1000 sec of stimulation (filled circles). B: Predicted mean synaptic weight lim_*t*→∞_〈*w*(*t*)〉 obtained from Eq 4. C,D: Same as A,B but for burst stimuli with three pulses per burst and an intraburst frequency of 120 Hz. Parameters: *A*_stim_ = 0.4, *f* = 10 Hz, *d*_e_ = 1 (A,B), and *A*_stim_ = 0.8, *f* = 5 Hz, *d*_e_ = 1, and an intraburst frequency of 120 Hz (C,D).

The PMCS-induced block structure of the synaptic weight matrix depended on the phase lags Δ*α*_1_ and Δ*α*_2_. Simulation results for the LIF network model are compared to theoretical predictions for the limit of long stimulation durations (lim_*t*→∞_〈*w*_*x*→*y*_(*t*)〉, Eq 1) in Fig 5A (single-pulse stimuli) and Fig 5C (burst stimuli with three pulses per burst). The long-term limit for weights of synapses in the blocks along the diagonal of the synaptic weight matrix—*intra-population synapses*—were insensitive to the phase lags Δ*α*_1_ and Δ*α*_2_. In contrast, the long-time limit of weights of synapses in the off-diagonal blocks—*inter-population synapses*—showed a pronounced dependence on the phase lags.

For the same spatio-temporal PMCS pattern, single-pulse and burst stimuli resulted in qualitatively different network structures. If the pattern illustrated in Fig 4A was delivered using single-pulse stimuli, all synaptic connections were down-regulated (Fig 4F). Thus, such PMCS led to a complete decoupling of the LIF neurons (〈*w*_*x*→*y*_〉 ≈ 0). In contrast, if the same PMCS pattern was delivered using burst stimuli with three pulses per burst, a qualitatively different synaptic network structure was obtained (Fig 4I). Intra-population synapses (diagonal blocks of the synaptic weight matrix) and synapses between subpopulations one and three were up-regulated, while synapses between other subpopulations were down-regulated. For the PMCS pattern illustrated in Fig 4A’, all but the synapses in the $W_{12}$ block were down-regulated during PMCS with single-pulse stimuli (Fig 4F’). In contrast, most synapses were up-regulated when burst stimuli were employed (Fig 4I’).

A more detailed analysis is presented in Fig 5. Here, simulation results are compared to theoretical estimates of the synaptic weight matrix after long stimulation durations. Single-pulse and burst stimuli resulted in qualitatively different network structures. Intra-population synapses were down-regulated for single-pulse stimuli (Fig 5A). In contrast, they were up-regulated for burst stimuli with three pulses per burst and an intraburst frequency of 120 Hz (Fig 5C). Furthermore, for the employed single-pulse stimuli and stimulation frequency, it was not possible to choose the phase lags Δ*α*_1_ and Δ*α*_2_ such that bidirectional coupling between different subpopulations was induced (up-regulation of both $W_{xy}$ and $W_{yx}$) (Fig 5A). However, this was possible for certain combinations of phase lags when burst stimuli were used instead. For instance, for Δ*α*_1/2_ ≈ 1 bidirectional coupling between subpopulations one and two was induced (Fig 5C).

We showed that the PMCS pattern and the shape of employed stimuli affected the stimulation-induced network structure. This suggests that PMCS can be tuned to induce a desired network structure, i.e., it may up-regulate certain synapses while down-regulating others.

### PMCS induces changes of the mean synaptic weight

We systematically varied the phase lags, Δ*α*_*k*_, characterizing the PMCS pattern, and analyzed the induced network structure at different times after stimulation onset. First, we focused our analysis on the mean synaptic weight, 〈*w*(*t*)〉 = ∑_*i*,*j*∈syn._ *w*_*i*→*j*_(*t*)/*N*_syn_. The sum runs over all synapses. The indices *i* and *j* refer to the presynaptic neuron and the postsynaptic neuron, respectively. *N*_syn_ is the total number of synapses in the network. Note that 〈*w*(*t*)〉 carries less information about the network structure than the block structure of the synaptic weight matrix. This is because different combinations of blocks of up- and down-regulated synaptic connections may result in similar values of 〈*w*(*t*)〉. Nevertheless an analysis of 〈*w*(*t*)〉 can uncover which PMCS patterns lead to the up-regulation (down-regulation) of a certain portion of these blocks.

Stimulation-induced changes of 〈*w*(*t*)〉 are of particular interest in the context of decoupling stimulation [59, 60]. Decoupling stimulation was suggested to down-regulate pathological synaptic connectivity to drive multistable plastic neuronal networks into the attractor of a stable weakly connected desynchronized state, such that desynchronization effects persist after cessation of stimulation. In our LIF network model, this would correspond to a weakening of excitatory connections [60, 61]. Of particular interest in this context are PMCS patterns that lead to a substantial reduction of 〈*w*(*t*)〉.

Theoretical predictions for 〈*w*(*t*)〉 after long stimulation durations for a single-pulse stimulus and a burst stimulus with three pulses per burst are presented in Fig 5B and 5D, respectively. lim_*t*→∞_〈*w*(*t*)〉 was invariant under the transformations (Δ*α*_1_, Δ*α*_2_) → (Δ*α*_2_, Δ*α*_1_) and (Δ*α*_1_, Δ*α*_2_) → (1 − Δ*α*_1_, 1 − Δ*α*_2_). These symmetries result from the homogeneous network structure, equally sized subpopulations, and the invariance of the mean synaptic weight under shuffling of the weight matrix’s components. Furthermore, lim_*t*→∞_〈*w*(*t*)〉 only depended on the phase lags modulo one as the addition of integers to the phase lags results in a shift of the PMCS pattern by multiples of the stimulation period, which did not affect 〈*w*(*t*)〉 after long stimulation durations if STDP was sufficiently slow.

Next, we compared the theoretical predictions for 〈*w*(*t*)〉 to simulation results for networks of LIF neurons with STDP. Prior to stimulation, the LIF network was prepared in a synchronized state with a mean synaptic weight of 〈*w*(*t*)〉 = *w*_0_. Thus, a reduction of 〈*w*(*t*)〉 below *w*_0_ indicates an overall decoupling whereas 〈*w*(*t*)〉 > *w*_0_ indicates overall strengthening of synapses. Note that stimulation may up-regulate certain groups of synapses while down-regulating others (Fig 5).

**Fig 6:**
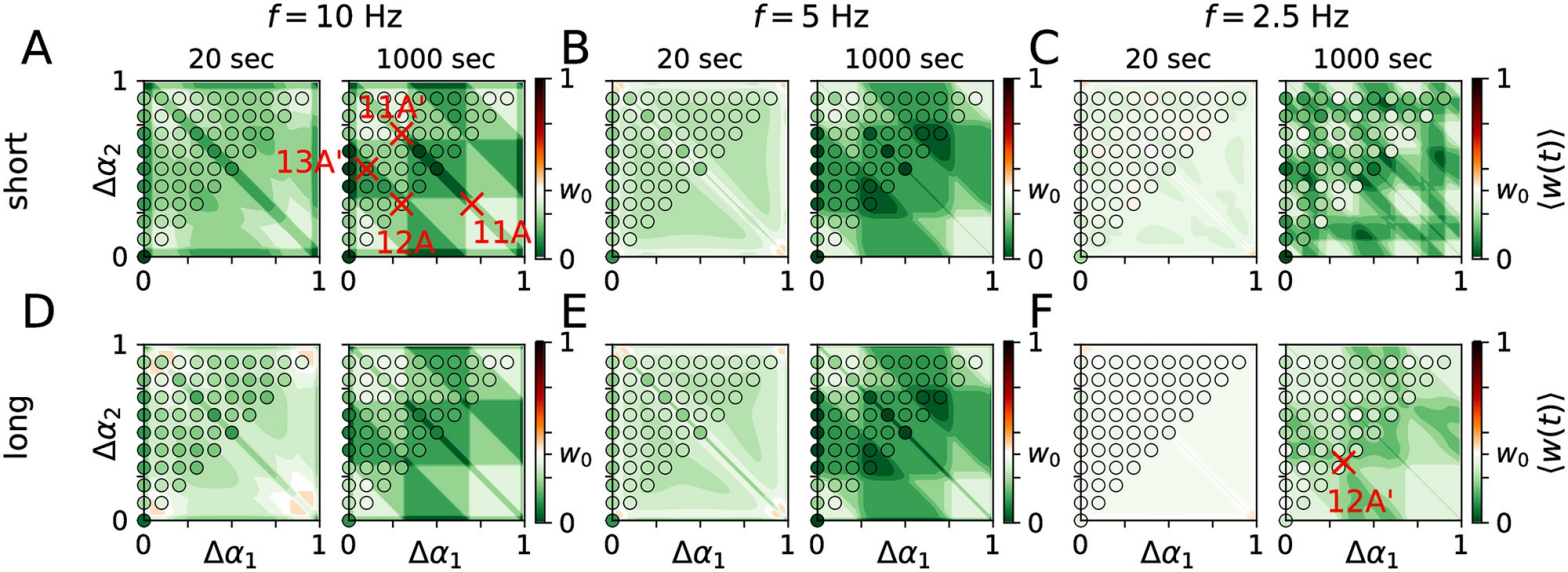
Mean synaptic weight during single-pulse PMCS depends on pulse duration and stimulation frequency. Theoretical predictions (color maps) obtained from Eq 4 after 20 sec and 1000 sec of PMCS are compared to simulation results for the LIF network model (filled circles). *w*_0_ = 0.38 is the mean synaptic weight prior to stimulation. Labeled columns show results for different stimulation frequencies: *f* = 10.0 Hz (A, D), *f* = 5.0 Hz (B, E), and *f* = 2.5 Hz (C, F). Rows show results for short pulses (*d*_e_ = 1, top) and long pulses (*d*_e_ = 20, bottom) (Methods). Simulated spike trains, snapshots of the connectivity matrix, and theoretical predictions of the block structure of the synaptic weight matrix are shown below for the parameter sets marked by red crosses (see red figure labels). Parameters: *A*_stim_ = 0.4.

Results for single-pulse stimuli with different pulse durations and PMCS frequencies are shown in Fig 6. Stimulation led to either a reduction or an increase of 〈*w*(*t*)〉 depending on the phase lags Δ*α*_1_ and Δ*α*_2_ and the pulse duration (Fig 6). PMCS with short single-pulse stimuli typically reduced 〈*w*(*t*)〉 more than PMCS with long single-pulse stimuli (compare simulation results in different rows of Fig 6). However, this effect was rather weak for the considered range of pulse durations.

Faster stimulation typically led to a faster reduction of 〈*w*(*t*)〉 as more stimuli per time were delivered (compare columns for *t* = 20 sec in Fig 6). The theoretical approximations for 〈*w*(*t*)〉 (Eq 4) are shown as color maps in Fig 6 and show good quantitative agreement with simulation results for fast stimulation and short pulses. For long pulses and fast stimulation, theoretical approximations accurately predicted 〈*w*(*t*)〉 after long stimulation, which depends on the signs of the mean rates of weight change for the individual synaptic populations rather than the exact values (Eq 1). Deviations occurred for long pulse durations and slow stimulation for which stimulus-triggered spiking responses were more susceptible to synaptic input from other subpopulations. This led to deviations between simulation results and theoretical predictions. The latter were derived under the assumption of stimulation-controlled spiking.

Next, we considered burst stimuli. As shown in Fig 2F, neurons fired multiple spikes in response to sufficiently strong burst stimuli. Raster plots of the spiking activity of LIF neurons during PMCS employing burst stimuli are shown in Fig 4G and 4G’, respectively.

**Fig 7:**
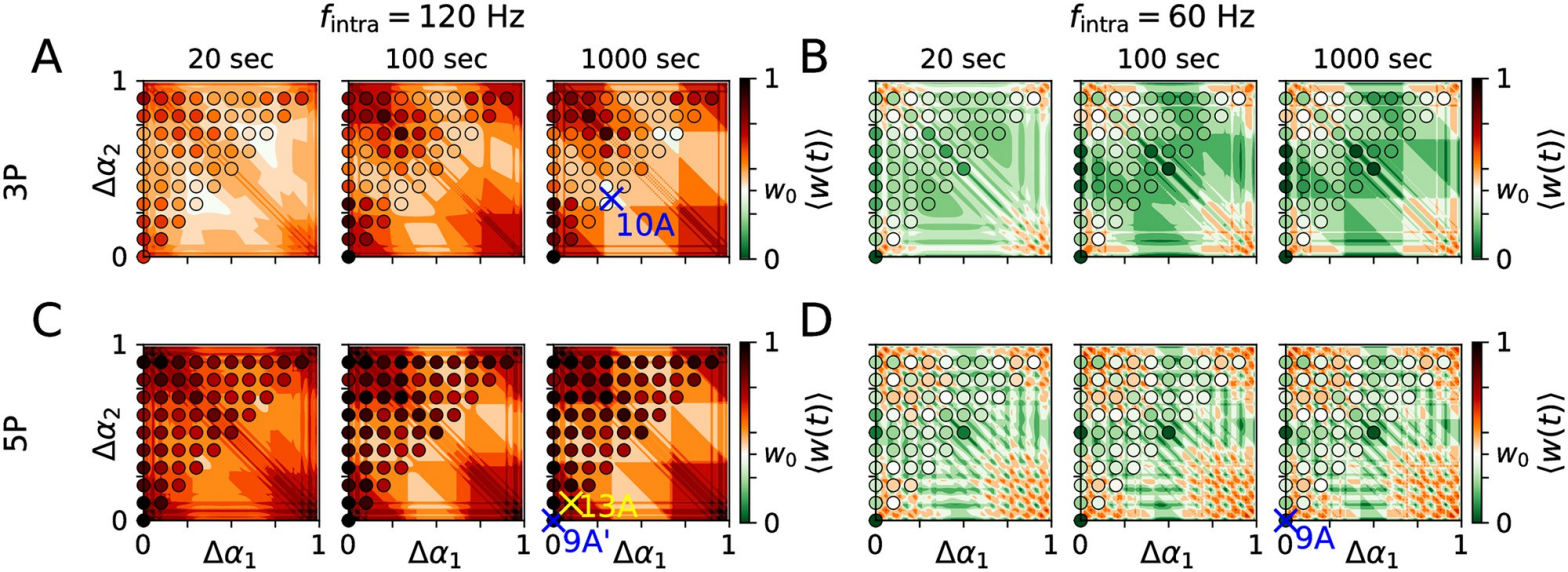
Mean synaptic weight during PMCS with burst stimuli depends on intraburst frequency and number of pulses per burst. Panels A,C and B,D show results for PMCS with burst stimuli with high (120 Hz) and low (60 Hz) intraburst frequencies, respectively. Rows show results for burst stimuli with three (top, 3P) and five (bottom, 5P) pulses, respectively. Columns show the mean synaptic weight 20 sec (left), 100 sec (middle), and 1000 sec (right) after onset of PMCS. Color maps show theoretical predictions, obtained from Eq 4, and filled circles resulted from simulations of the LIF network model. Simulated spike trains, snapshots of the synaptic weight matrix, and corresponding theoretical predictions of the block structure of the synaptic weight matrix, obtained from Eq 1, are shown below for the parameter sets marked by colored crosses (see corresponding figure labels). Parameters: *f* = 5 Hz, *A*_stim_ = 0.8, *d*_e_ = 1.

Results for 〈*w*(*t*)〉 during PMCS with burst stimuli are shown in Fig 7. 〈*w*(*t*)〉 depended on the phase lags, Δ*α*_1_ and Δ*α*_2_. Furthermore, the shape of burst stimuli strongly affected 〈*w*(*t*)〉. For bursts with large numbers of pulses, small variations of the phase lags strongly affected 〈*w*(*t*)〉 (Fig 7). This parameter sensitivity was most pronounced for phase lags that where close but not equal to zero (modulo one) (panel corners in Fig 7).

In a portion of the (Δ*α*_1_,Δ*α*_2_)-plane, PMCS with burst stimuli with a large number of pulses led to larger 〈*w*(*t*)〉 than PMCS with single-pulse or burst stimuli with low numbers of pulses (compare Figs 6 and 7). This effect was most pronounced in the panel corners, where phase lags were small but non-zero and even the reduction of 〈*w*(*t*)〉 by PMCS with single-pulse stimuli was weak (see Fig 6).

Not only the number of pulses per burst but also the intraburst frequency affected 〈*w*(*t*)〉. Low intraburst frequencies typically led to smaller 〈*w*(*t*)〉 than high intraburst frequencies (compare Fig 7A and 7C with Fig 7B and 7D).

### PMCS-induced decoupling

Motivated by a recent study on decoupling stimulation [60], we analyzed to which extend PMCS may be employed as decoupling stimulation, i.e., was able to reduce the mean synaptic weight substantially. We found that the decoupling properties of PMCS depended on the phase lags, the stimulation frequency, and the stimulus type.

In Figs 6 and 7, fast PMCS employing single-pulse stimuli led to an overall reduction of the mean synaptic weight across the (Δ*α*_1_, Δ*α*_2_)-plane (Fig 6A, 6B and 6D). We also observed a reduction of the mean synaptic weight for burst stimuli with low intraburst frequency; however, this reduction was sensitive to the phase lags Δ*α*_1_ and Δ*α*_2_ (Fig 7B and 7D). For both stimuli, the fastest reduction of the mean synaptic weight was obtained for either periodic stimulation (Δ*α*_1/2_ = 0) or for PMCS patterns where two subpopulations received stimuli simultaneously, i.e., Δ*α*_2_ ≈ 1 − Δ*α*_1_. We also want to highlight the PMCS pattern with Δ*α*_1/2_ = 1/*M*, which corresponds to the original CRS pattern with fixed sequence introduced in Refs. [17, 19]. For these three types of PMCS patterns, we analyzed how the mean synaptic weight during stimulation depended on the stimulation frequency and the stimulus type.

**Fig 8:**
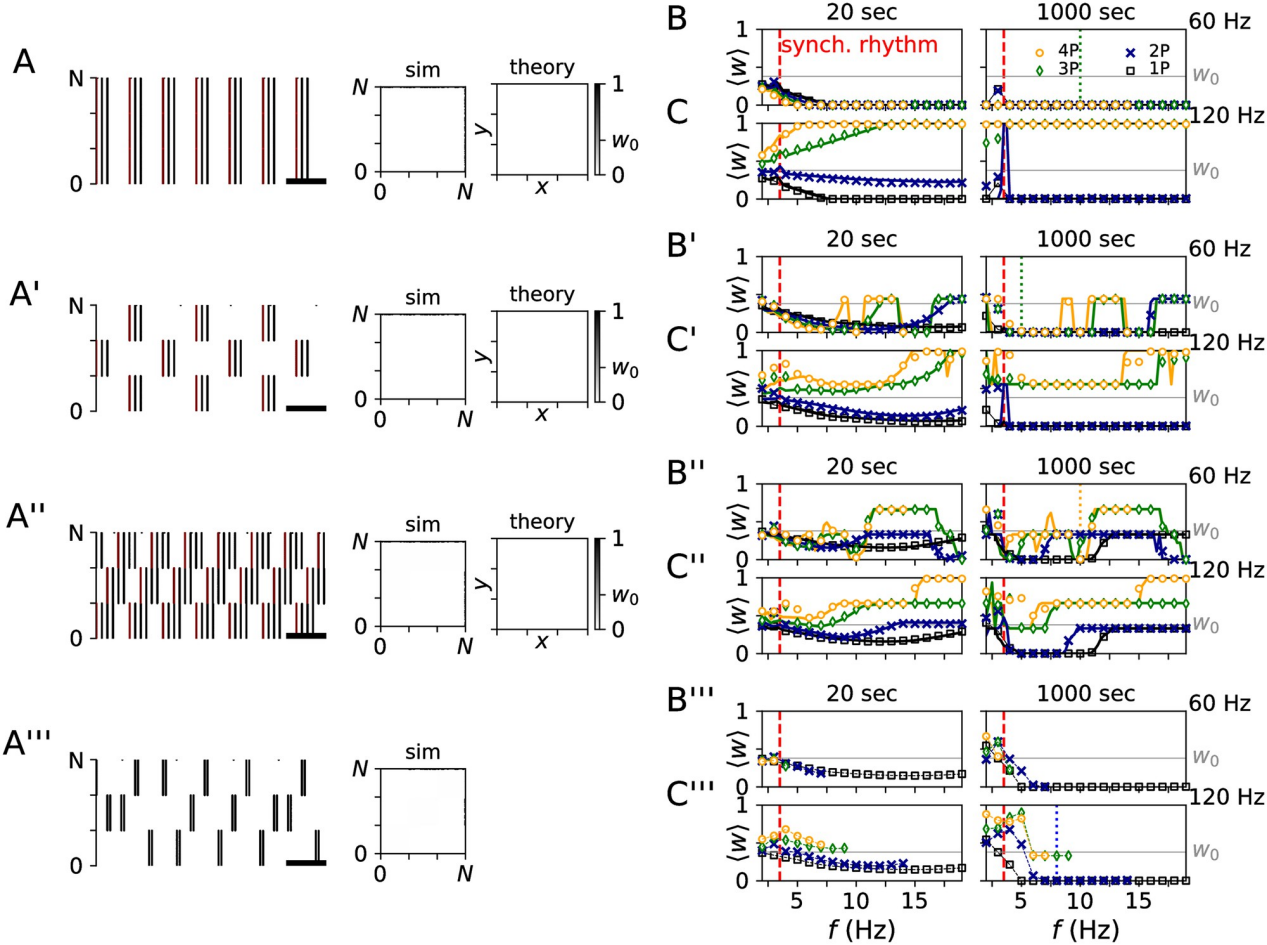
Decoupling effects of three PMCS patterns compared to decoupling by CRS RVS. A: Raster plot of spiking activity of LIF neurons after 1000 sec of periodic stimulation (Δ*α*_1/2_ = 0) using burst stimuli of three pulses per burst and an intraburst frequency of 60 Hz. Furthermore, snapshots of the synaptic weight matrix of the LIF network (sim) and the corresponding theoretical approximation obtained from Eq 1 (theory) are shown. Successful stimulation-induced decoupling led to synaptic weights that were close to zero (white). B, C: The mean synaptic weight after 20 sec and 1000 sec of periodic stimulation as function of the stimulation frequency, *f*, for single-pulse stimuli (1P) and burst stimuli with two (2P), three (3P), and four (4P) pulses per burst (see legend). Panels B and C show results for an intraburst frequency of 60 Hz (B) and 120 Hz (C), respectively. Markers show simulation results (averaged over five network realization) and curves theoretical approximations (Eq 4). Vertical lines mark the frequency of the synchronous rhythm prior to stimulation (red dashed line) and the set of stimulation parameters corresponding to the raster plot shown in panel A (colored dotted line), respectively. A’-C’ and A”-C”: Same as A-C but for PMCS with Δ*α*_1/2_ = 0.5 (A’-C’) and Δ*α*_1/2_ = 0.33 (A”-C”), respectively. (a’’’-c’’’): Similar plots for CRS RVS. Parameters: *d*_e_ = 1 (all panels). *A*_stim_ = 0.8 for PMCS with burst and CR RVS with single-pulse and burst stimuli. *A*_stim_ = 0.4 for PMCS with single-pulse stimuli (B, C, B’, C’, and B”, C”).

In Fig 8, we compare simulation results for the network of LIF neurons to theoretical predictions for the mean synaptic weight obtained from Eq 4. Complete decoupling, 〈*w*(*t*)〉 ≈ 0, was possible for all three PMCS patterns if the stimulus type and the stimulation frequency were adjusted accordingly.

For periodic stimulation, a complete decoupling was achieved for single-pulse stimuli and for burst stimuli with two pulses per burst for a wide range of stimulation frequencies (Fig 8A, 8B and 8C). For bursts with more pulses, decoupling was only obtained for a low intraburst frequency (60 Hz, Fig 8B). In contrast, for a high intraburst frequency (120 Hz), burst stimuli with more pulses per burst led to an increase of the mean synaptic weight during stimulation. The fastest decoupling was achieved by PMCS with burst stimuli and a low intraburst frequency (Fig 8B, panel for *t* = 20 sec).

For the PMCS pattern with Δ*α*_1/2_ = 0.5, complete decoupling was obtained over a wide frequency range if either single-pulse stimuli or burst stimuli with two pulses per burst and high intraburst frequency were used (Fig 8C’). For other stimuli, we found decoupling only in finite frequency intervals. For other stimulation frequencies, the mean synaptic weight approached non-zero values (see Fig 8B’ and 8C’). Thus, the decoupling effects of this PMCS pattern were less robust with respect to variations of the stimulation frequency than those of periodic stimulation.

For the PMCS pattern with Δ*α*_1/2_ = 1/*M*, decoupling was even less robust with respect to changes of the stimulation frequency. For high intraburst frequencies, we only found decoupling for single-pulse stimuli and burst stimuli with two pulses per burst and only in limited frequency intervals (Fig 8C”). For low intraburst frequencies, it was possible to fine-tune the stimulation frequency such that complete decoupling was achieved. Overall, complete decoupling occurred for less frequencies the more pulses per burst stimulus were used (Fig 8B”). PMCS employing other stimuli typically led to non-zero mean synaptic weight (Fig 8B” and 8C”).

For comparison, we also performed simulations for CRS with rapidly varying sequence (CRS RVS). During CRS RVS each subpopulation receives exactly one stimulus during a cycle period of duration 1/*f*. In contrast to CRS with fixed sequence, the sequence at which individual subpopulation receive stimuli is shuffled after each cycle period (see Fig 8A’’’). CRS RVS has been used in preclinical and clinical studies on CRS as a treatment for PD [20, 23]. Its decoupling properties for single-pulse stimuli have been analyzed in great detail in Ref. [61]. For single-pulse stimuli, CRS RVS led to decoupling for a wide frequency range (Fig 8B’’’ and 8C’’’). Its decoupling effects were more robust with respect to changes of the stimulation frequency than those of PMCS with Δ*α*_1/2_ = 1/*M*, which corresponds to CRS with fixed sequence (compare Fig 8 panels B”, C” and B’’’, C’’’). However, besides single-pulse stimuli, only burst stimuli with two pulses per burst led to a complete decoupling in the LIF network. Note that only a limited range of stimulation frequencies was studied for CRS RVS in order to avoid an overlap of stimuli delivered to the same subpopulation.

### PMCS-induced manipulation of synaptic network structure

Of particular interest is how PMCS can be employed to induce a desired network structure, i.e., to down-regulate certain synaptic populations while up-regulating others. Following, we study how the PMCS parameters can be adjusted to modulated intra- and inter-population synapses such that a particular synaptic network structure is induced.

#### Stimulus shape determines dynamics of intra-population synapses

In an earlier study, it was suggested that the dynamics of intra-population synapses is strongly affected by the shape of administered stimuli [60]. This effect was referred to as *stimulus-induced reshaping* of synaptic connectivity. In order to study the impact of the stimulus shape on the synaptic weight dynamics of intra-population synapses, we considered the case of periodic stimulation, i.e. Δ*α*_1/2_ = 0 (modulo one). During periodic stimulation all synapses can be considered as intra-population synapses as the entire neuronal population receives stimuli simultaneously.

First, we considered single-pulse stimuli. Differences between periodic stimulation employing either short or long single-pulse stimuli can be inferred from the data points for Δ*α*_1/2_ = 0 in Fig 6. Short single-pulse stimuli often led to faster decoupling, i.e. a faster down-regulation of intra-population synapses, than long single-pulse stimuli (data points for *t* = 20 sec in Fig 6).

Next, we compared results for single-pulse and burst stimuli with different numbers of pulses per burst. Corresponding simulation results are compared to theoretical approximations in Fig 8B and 8C. The dynamics of the mean synaptic weight strongly depended on the intraburst frequency. Specifically, for low intraburst frequencies bursts with more pulses per burst led to faster decoupling (Fig 8B, panel for *t* = 20 sec). The opposite trend occurred for high intraburst frequencies. Here, only single-pulse stimuli and bursts with two pulses led to a reduction of the mean weight whereas more pulses per bursts led to a strengthening of synapses.

**Fig 9:**
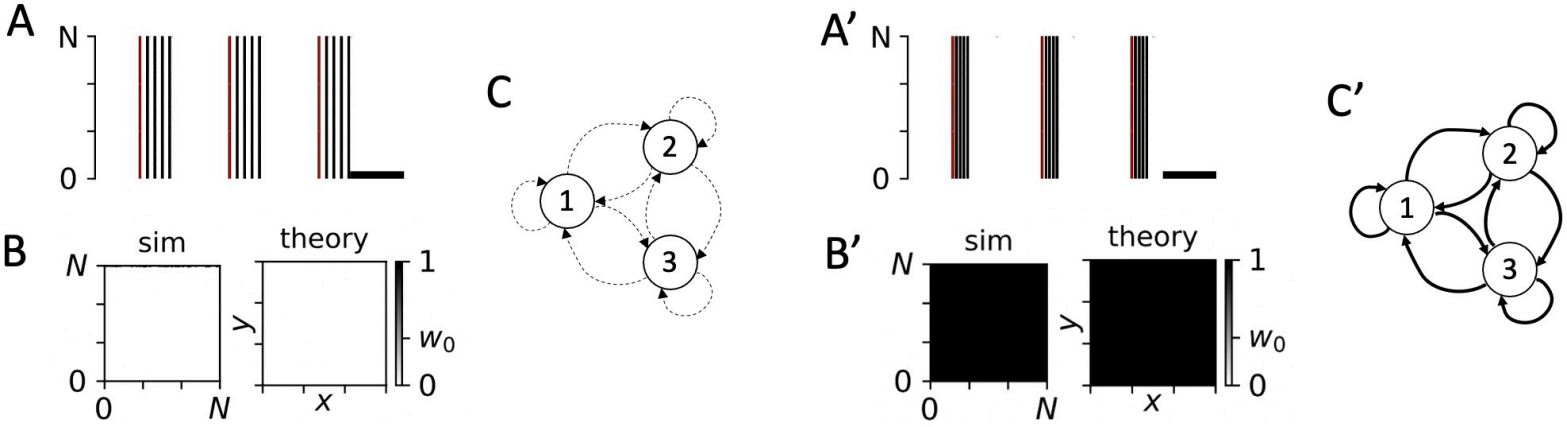
Periodic stimulation may either up-regulate or down-regulate all synapses depending on the employed type of stimuli. A: Raster plot of simulated spiking activity for PMCS with Δ*α*_1/2_ = 0 and burst stimuli with low intraburst frequency. The scale bar indicates a time window of 100 ms. Stimuli are illustrated by red blocks. B: Snapshots of the simulated synaptic weight matrix after 1000 sec of stimulation (sim) and the corresponding theoretical prediction obtained from Eq 1 (theory). C: Schematic of stimulation-induced network structure. Circles represent subpopulations one, two, and three. Dashed arrows illustrate down-regulated synaptic connections. A’-C’: Same as A-C but for burst stimuli with high intraburst frequency. Thick arrows in panel C’ illustrate up-regulated synaptic connections. Parameters: *A*_stim_ = 0.8, *f* = 5 Hz, and *d*_e_ = 1 (all panels). An intraburst frequency of 60 Hz and five pulses per burst were used in panels A-C, and an intraburst frequency of 120 Hz and five pulses per burst were used in panels A’-C’.

Thus, periodic stimulation may either lead to a down-regulation of all (intra-population) synapses or to an up-regulation of all (intra-population) synapses, depending on the shape of employed stimuli. If burst stimuli are used, the intraburst frequency plays a key role for the dynamics of (intra-population) synapses: low intraburst frequencies result in a down-regulation of synapses, whereas high intraburst frequencies result in an up-regulation. Raster plots of exemplary spiking activity and snapshots of the synaptic weight matrix for the network of LIF neurons during periodic stimulation with two types of burst stimuli are shown in Fig 9.

#### PMCS pattern determines the dynamics of inter-population synapses

**Fig 10:**
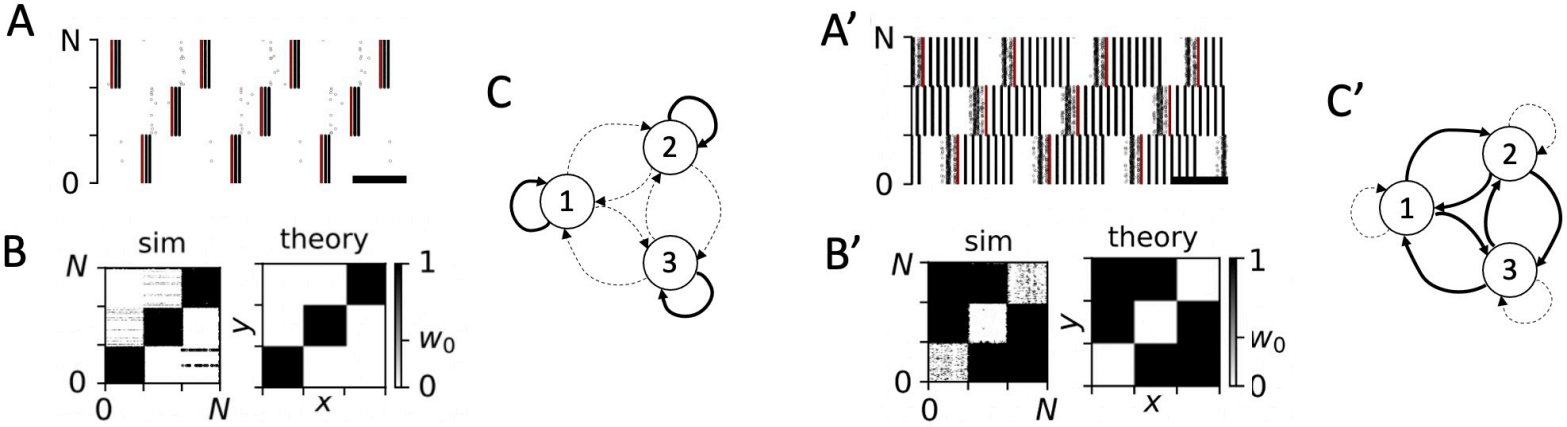
PMCS can be employed to modulate intra- and inter-population synapses in opposite ways. A: Raster plot of simulated spiking activity for PMCS with burst stimuli with high intraburst frequency. B: Snapshots of simulated synaptic weight matrix after 1000 sec of stimulation (sim) and the corresponding theoretical prediction obtained from Eq 1 (theory). C: Schematic of stimulation-induced network structure (see caption of Fig 9 for symbols). A’-C’: Same as A-C but for burst stimuli with low intraburst frequency. Parameters: *A*_stim_ = 0.8, *f* = 5 Hz, and *d*_e_ = 1 (all panels). In panels A-C, we used an intraburst frequency of 120 Hz, three pulses per burst, and Δ*α*_1/2_ = 0.33 (A-C), In panels A’-C’, we used 60 Hz, eight pulses per burst, and Δ*α*_1/2_ = 0.31 (A’-C’).

**Fig 11:**
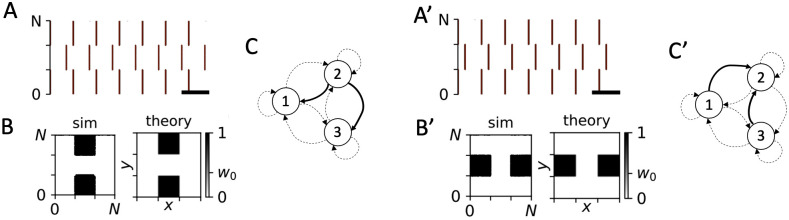
PMCS-induced tree and reverse-tree network structures. A: Raster plot of the spiking activity in the LIF network model 1000 sec after onset of PMCS with single-pulse stimuli and small positive phase lags between stimuli delivered to subpopulation two and subpopulations one and three. B: A snapshot of the synaptic weight matrix after 1000 sec of stimulation (sim) and the corresponding theoretical prediction obtained from Eq 1 (theory). C: Schematic of stimulation-induced tree network structure (see caption of Fig 9 for symbols). A’-C’: Same as A-C but for single-pulse PMCS with small negative phase lags between stimuli delivered to subpopulation two and subpopulations one and three. Parameters: *A*_stim_ = 0.4, *f* = 10 Hz, and *d*_e_ = 1 (all panels) and Δ*α*_1_ = 0.7, and Δ*α*_2_ = 0.3 (A-C), and Δ*α*_1_ = 0.3, and Δ*α*_2_ = 0.7 (A’-C’).

**Fig 12:**
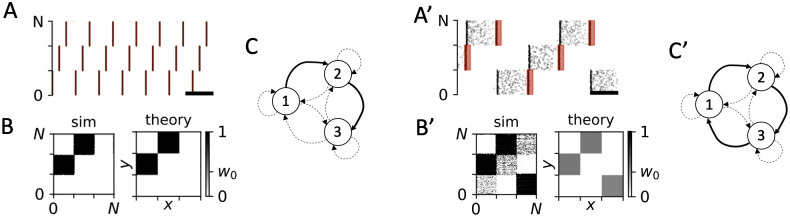
PMCS-induced feed-forward and circular network structure. A: Raster plot of simulated spiking activity in the LIF network model 1000 sec after onset of PMCS with single-pulse stimuli and small positive phase lags between subsequent subpopulations. B: Snapshots of simulated synaptic weight matrix after 1000 sec of stimulation (sim) and the corresponding theoretical prediction obtained from Eq 1 (theory). C: Schematic of stimulation-induced feed-forward structure (see caption of Fig 9 for symbols). A’-C’: Same as A-C but for slow PMCS with long single-pulse stimuli. The phase lags were adjusted to cause a circular network structure. Parameters: *A*_stim_ = 0.4 and Δ*α*_1/2_ = 0.3 (A-C), and Δ*α*_1/2_ = 0.33 (A’-C’) and *d*_e_ = 1 and *f* = 10 Hz (A-C), and *d*_e_ = 20 and *f* = 2.5 Hz (A’-C’).

**Fig 13:**
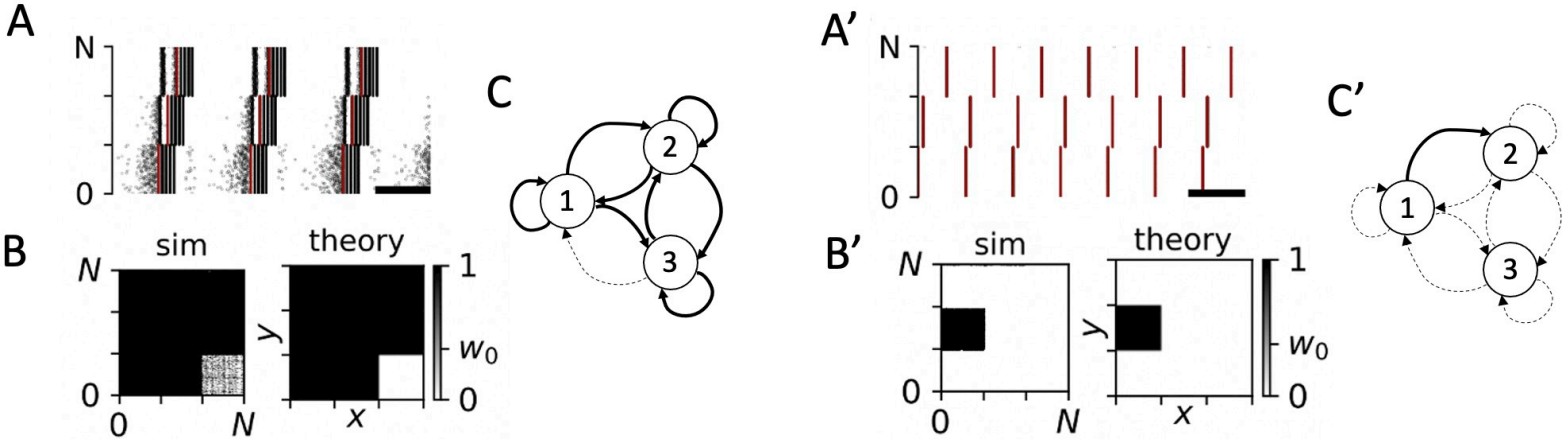
PMCS-induced network structures in which either all-but-one or only one synaptic population was up-regulated while all others were down-regulated. A: Raster plot of simulated spiking activity of LIF neurons 1000 sec after onset of PMCS with burst stimuli with high intraburst frequency and short phase lags between subsequent subpopulations. B: Snapshots of simulated synaptic weight matrix after 1000 sec of stimulation (sim) and the corresponding theoretical prediction obtained from Eq 1 (theory). C: Schematic of stimulation-induced network structure (see caption of Fig 9 for symbols). A’-C’: Same as A-C but for single-pulse PMCS. One phase lag was chosen to be significantly smaller than the others in order to up-regulate only one population of synapses. Parameters: *d*_e_ = 1 (all panels). In panels A-C, we used *A*_stim_ = 0.8, an intraburst frequency of 120 Hz, five pulses per burst, *f* = 5 Hz, and Δ*α*_1/2_ = 0.1. In panels A’-C’, we used single-pulse stimuli with *A*_stim_ = 0.4, a stimulation frequency of *f* = 10 Hz, and the phase lags Δ*α*_1_ = 0.1 and Δ*α*_2_ = 0.5.

Next, we analyzed the dynamics of weights of inter-population synapses. To this end, we chose non-zero phase lags (Δ*α*_*k*_ ≠ 0) between stimulus trains delivered to adjacent neuronal subpopulations. We tuned the phase lags to induce several network structures in the network of LIF neurons. We present the results for the following network structures:

- **Networks with up-regulated intra- and down-regulated inter-population synapses (and vice versa)**. We employed PMCS to modulate intra- and inter-population synapses in opposite ways. Applying our results from the previous paragraphs, the dynamics of intra-population synapses (diagonal blocks of the synaptic weight matrix) can be controlled by the stimulus shape. We used burst stimuli with high intraburst frequency to up-regulate and burst stimuli with low intraburst frequency to down-regulate intra-population synapses. With adjusted phase lags of approximately 1/3, PMCS led to network structures in which intra-population synapses were up-(down-)-regulated whereas inter-population synapses were down-(up-)regulated. Especially for burst stimuli, the stimulation frequency had a strong impact on the stimulation-induced network structure, as overlapping burst stimuli led to complex synaptic weight dynamics of inter-population synapses. Simulated spike trains, synaptic weight matrices, and schematics of the stimulation-induced network structures are presented in Fig 10.
- **Tree and reverse-tree networks**. We tested whether PMCS can induce a tree or reverse-tree network structure in the LIF network. In these network structures, one subpopulation provides input to the other two subpopulations (tree) or receives input from these subpopulations (reverse-tree). As inducing these network structures required the down-regulation of intra-population synapses, we used single-pulse stimuli. Furthermore, our results for two subpopulations (Fig 3) showed that short positive phase lags between subpopulations lead to an up-regulation, whereas short negative or long positive phase lags lead to a down-regulation of synapses interconnecting subpopulations. We chose Δ*α*_2_ = 1 − Δ*α*_1_ (modulo one) with small negative Δ*α*_1_ for a tree structure where subpopulation two provides input to subpopulations one and three and small positive Δ*α*_1_ for a reverse-tree network structure. Corresponding simulation results are presented in Fig 11A, 11B and 11C for the tree network structure and in Fig 11A’, 11B’ and 11C’ for the reverse-tree network structure.
- **Feed-forward and circular networks**. Using small positive phase lags between subsequent neuronal subpopulations allowed us to induce a feed-forward network structure (Fig 12A, 12B and 12C) and a circular network structure (see Fig 12A’, 12B’ and 12C’). In more detail, phase lags should be chosen such that up-regulation is achieved only for inter-population synapses between subsequent subpopulations (see Fig 3 for possible combinations of stimulation parameters).
- **Up- or down-regulation of one synaptic population**. Lastly, we tuned the PMCS pattern such that a single population of synapses was affected in the opposite way as all others. To down-regulated only one population of synapses while up-regulating the others, burst stimuli with high intraburst frequency were used. Corresponding simulation results are shown in Fig 13A, 13B and 13C. To up-regulate one population of synapses while down-regulating the others, short single-pulse stimuli were used (see Fig 13A’, 13B’ and 13C’). Note that it was not possible to modulate a single population of intra-population synapses differently than the others. For such a manipulation, one would have to deliver different stimuli to the individual subpopulations, which we did not consider in the present paper.

## Discussion

Several neurological disorders are associated with impaired synaptic connectivity. We suggest PMCS to harness synaptic plasticity to counteract pathological synaptic connectivity. During PMCS, phase-shifted periodic stimulus trains are administered to different neuronal subpopulations. We studied synaptic reshaping during PMCS employing single-pulse and burst stimuli in networks of excitatory LIF neurons with STDP. We showed that: (a) The phase lags between stimulus trains can be tuned to up-regulate certain populations of synapses while down-regulating others. This way, a desired network structure can be induced. (b) The type of administered stimuli strongly affects the PMCS-induced network structure. (c) During PMCS with burst stimuli an adjustment of the intraburst frequency allows for either up- or down-regulation of synapses between neurons within the same subpopulation. (d) PMCS can be employed as a decoupling stimulation. In particular, PMCS patterns in which multiple subpopulations receive stimuli simultaneously led to decoupling effects that were robust with respect to changes of the stimulation frequency if appropriate stimuli were used. In our LIF model such stimuli included single-pulse stimuli and burst stimuli with low intraburst frequency. Our computational analysis of PMCS in networks of LIF neurons and our theoretical approximations revealed stimulation strategies for a controlled modulation of synaptic connectivity. PMCS may be suitable to counteract pathological synaptic connectivity in patients suffering from neurological diseases and may present a step towards novel treatments that lead to symptom relief that outlasts stimulation.

### Combined computational and theoretical approach to predict PMCS-induced synaptic reshaping

To predict synaptic reshaping during PMCS, we presented a combined computational and theoretical approach, consisting of the following steps. (i) The statistics of stimulus-triggered neuronal spikes were estimated based on simulations of LIF neurons during periodic stimulation of one neuronal subpopulation. (ii) These statistics were used to estimate the mean rate of synaptic weight change during stimulation; and (iii), the latter estimate was used to predict the dynamics of the weights of synapses between individual neuronal subpopulations.

Steps (i) and (ii) of our approach result in estimates of the mean rate of weight change. These estimates were compared to simulation results for the LIF network model in which two subpopulations received stimuli at a fixed phase lag, which resembled a standard STDP experiment [35]. The theoretical predictions approximated the simulation results well for fast stimulation for various stimulus types (Fig 3). Deviations for slow stimulation resulted from the assumption that the distribution of stimulus-triggered spikes does not change during PMCS, made during the derivation of Eq 19. This implicitly assumed weak synaptic interaction and strong stimulation. Accordingly, deviations of simulation results from our theoretical predictions occurred if spiking responses were perturbed by synaptic input from other subpopulations, mainly when this input resulted from stimulus-triggered collective spiking events (see, for instance, Fig 12A’, 12B’ and 12C’). During slow stimulation, the LIF neurons were close to their spiking thresholds for a substantial part of the ISIs. This made them more susceptible to synaptic input from other neuronal subpopulations. For burst stimuli with large numbers of pulses per burst, individual stimulus pulses triggered sharp collective spiking responses. These led to strong synaptic input to other neuronal subpopulations, which led to deviations (Figs 10A’, 10B’, 10C’, 13A, 13B and 13C).

An alternative to estimating the mean rate of weight change based on the statistics of stimulus-triggered spikes (steps (i) and (ii)) would be to estimate the mean rate of weight change experimentally or from simulation data (just as we did in Fig 3). Then, these estimates can be used in step (iii) (Eq 1) to predict the outcome of PMCS in experiments or other computational models.

Our approach can be applied to a wide range of computational neuron and network models. Furthermore, PMCS delivered to two subpopulations corresponds to a standard STDP experiment, where postsynaptic and presynaptic spikes are elicited by applying phase-shifted stimulus trains [34, 35]. The case of two subpopulations also applies to experiments on paired associative stimulation during which repetitive low-frequency median nerve stimulation was paired with transcranial magnetic stimulation (TMS) [68–70]. This idea has been extended to pair different types of stimuli in order to study synaptic plasticity in different brain areas. For instance, DBS stimuli were paired with cortical TMS stimuli in Refs. [15] and [16]. This suggests that our results may apply to various types of brain stimulation, including DBS, vibrotactile fingertip stimulation, and TMS.

In the present paper, we applied our approach to networks of excitatory LIF neurons with STDP. While the LIF neurons responded with high fidelity to administered stimuli, our general approach assumes that neuronal spiking responses are entrained with the periodic stimulus trains. Such an entrainment of oscillators with periodic stimulation is a general phenomenon and has been observed in various biological systems [71]. For instance, the entrainment of neuronal spike rhythms to HF DBS was studied in target areas for the treatment of PD, i.e., in the basal ganglia and the thalamus. There, it was observed that neuronal activity in brain areas that received synaptic input from the stimulated target brain regions entrained with the stimulation [72, 73]. Individual spike times occurred at fixed phases of the HF DBS rhythm, with millisecond precision [72, 73]. Furthermore, an entrainment of cortical activity to HF DBS was observed, suggesting antidromic activitation of cortical neurons by STN HF DBS [74]. Besides DBS, several other stimulation techniques were found to cause an entrainement of neuronal activity with administered stimuli. For instance, during vibrotactile stimulation an entrainment of neuronal spiking responses with skin indentation oscillations was observed in the human somatosensory thalamus [75] and in the monkey primary somatosensory cortex [76, 77]. An entrainment of neuronal activity with stimulus deliveries was also reported by experimental studies on TMS [78] and transcranial alternating current stimulation [79], as well as visual stimulation [80]. In Ref. [81], tremor entrainment was achieved by periodic burst stimulation. Their computational results suggested that this was caused by an entrainment of tremor-related neurons with the stimulus pattern. Widely observed entrainment of neuronal activity with different types of periodic brain stimulation suggests that PMCS may be delivered using a variety of stimulation techniques and that our combined computational and theoretical approach may be applicable.

### Statistics of stimulus-triggered spikes and synaptic plasticity determine synaptic reshaping

We delivered PMCS to two neuronal subpopulations. Our analysis revealed a strong dependence of the mean rate of synaptic weight change on stimulation parameters (Fig 3). Applying our combined computational and theoretical approach, we were able to explain this dependency by considering the impact of stimulation parameters on the statistics of stimulus-triggered spikes and calculating the resulting mean rate of synaptic weight change in neuronal networks with STDP.

A strong dependence of synaptic reshaping on the statistics of stimulus-triggered neuronal responses has been observed experimentally. For instance, in the rat STN, Wang et al. observed that stimulus-triggered rebound bursts with more than two spikes per burst triggered long-term potentiation (LTP) of inhibitory synapses on STN neurons whereas bursts with two or less spikes caused long-term depression (LTD) or no change [82]. In another study on cortical neurons, a strong dependence of synaptic plasticity on the neuronal firing rate was observed [83].

Several types of synaptic plasticity have been observed in the brain. In the LIF network model and in our theoretical derivation of the mean rate of weight change, we considered a nearest-neighbor STDP scheme [84]. This presents a simplification as experimental studies found that the presynaptic and postsynaptic spike patterns affect the dynamics of synaptic weights [31, 36]. In general, weight updates during fast spike patterns, such as bursting, may not simply result from a superposition of weight updates for single spike pairs. One suggestion of how to account for this observation was to use STDP schemes that only consider pairings with the latest/next spike, so-called nearest-neighbor schemes [83, 84], comparable to the one we use in the present paper. Other studies suggested more complex multi-spike STDP schemes in which weight updates depend on the timing of triplets or quadruplets of spikes [85, 86].

In addition to the considered spike times, the effect of STDP strongly depends on the STDP function (Eq 10). An overview of experimentally observed STDP functions can be found in Ref. [87]. In our LIF network model, we considered canonical STDP [35, 67, 88] (see Fig 1C). Canonical STDP has been found in several brain regions including the neocortex [88], the hippocampus [35], and the striatum, where STDP was also modulated by dopamine [89].

While detailed information on the shape of the STDP function is often not available, several experimental and clinical studies reported brain stimulation-induced synaptic reshaping and long-lasting effects. In the context of PD, direct and indirect evidence for synaptic reshaping due to stimulation of target brain areas has been presented. Common target areas for therapeutic DBS in PD patients include the STN [90] and the internal segment of the globus pallidus (GPi) [91]. Stimulation-induced reshaping of synaptic connections in response to STN stimulation was observed in rat brain slices [10, 11, 14, 92]; however, while synaptic reshaping depended on the delivered stimulus pattern [11], to the best of our knowledge, only one study showed a dependence on the relative timings of postsynaptic and presynaptic spikes in these brain areas [14] and thereby provided evidence for STDP. Indirect evidence for STN stimulation-induced synaptic reshaping was presented by a study in PD patients [15]. There, STN DBS induced motor-cortical plasticity. In experiments STN DBS was paired with TMS of the primary motor cortex. Repetitive stimulation entailed long-lasting (≈ 45 min) changes of the amplitudes of motor evoked potentials (MEPs) [15]. Another study analyzed the effect of GPi DBS in a similar setup. Single DBS pulses were paired with motor-cortical TMS at different time lags [16]. The authors reported that repetitive delivery of paired stimuli entailed long-lasting changes (≈ 1 hour) of the amplitude of MEPs. Another line of indirect evidence indicates stimulation-induced synaptic reshaping during CRS. Long-lasting effects of CRS were observed in experiments in rat brain slices [93]; preclinical studies in MPTP monkeys, where CRS was delivered using down-scaled multisite DBS electrodes [20–22]; and in PD patients, where CRS was delivered through standard multisite DBS electrodes [23]. Recently, long-lasting therapeutic effects in PD patients were also induced by vibrotactile fingertip CRS [25, 26, 94]. There, multiple neuronal subpopulations were targeted by delivering phase-shifted stimuli to different fingertips. Neurons that respond to signals from cutaneous receptor afferents of different fingers are arranged somatotopically in the sensory cortex [95–97], which provides evidence that the resulting neuronal responses occurred primarily in segregated neuronal subpopulations related to input from the respective stimulated fingertips. Plasticity has also been induced using other types of brain stimulation. For instance, when phase-shifted stimuli were delivered to the two cortical hemispheres using transcranial alternating current stimulation [98]. The authors observed long-lasting changes in cortical connectivity. These changes were later described by a corresponding computational model incorporating STDP [99]. These lines of evidence suggest that various types of brain stimulation may indeed be suitable to realize the PMCS approach and induce long-lasting changes of synaptic connectivity. This may provide a tool to induce long-lasting therapeutic effects in patients suffering from neurological disorders associated with pathological synaptic connectivity.

### Stimulus shape impacts synaptic weight dynamics

Of particular interest is the impact of the stimulus shape on the synaptic weight dynamics, which was referred to as *stimulus-induced synaptic reshaping* in Ref. [60]. Our combined computational and theoretical approach revealed that changes of the stimulus type had a similar effect on the mean rate of weight change as if the same stimulus was delivered to networks with different STDP functions (see Fig 3). Consequently, the type of employed stimuli had a strong impact on the PMCS-induced network structure. For instance, in Fig 4, the same PMCS pattern delivered using either single-pulse or burst stimuli induced qualitatively different network structures.

Previous computational studies considered different stimuli during the delivery of CRS and studied long-lasting effects of stimulation. In Ref. [56], CRS was delivered using three different types of model stimuli: (a) bursts of charge-balanced electrical pulses, (b) a single excitatory postsynaptic potential (corresponding to stimulation of an incoming fiber of an excitatory neuron), and (c) a single inhibitory postsynaptic potential (stimulation of an incoming fiber of an inhibitory neuron). Case (a) was modeled by delivering electrical current pulses to stimulated neurons, similar to the electrical pulses considered here. The authors found that all three types of stimuli led to long-lasting desynchronization [56]. In their model, all three types of stimuli led to a phase reset of the stimulated neuronal subpopulation, which presumably resulted in a sharp distribution of stimulus-triggered spikes. Note that this distribution was not studied in detail; however, phase resetting was demonstrated numerically. Therefore, we speculate that the resulting distributions of stimulus-triggered spikes were rather similar even though different stimuli were used. Other computational studies delivered CRS using vibrotactile stimulation (see results for the computational model in [26]). Vibrotactile stimuli led to a broad distribution of stimulus-triggered spikes. Their results differed significantly from results for electrical model stimuli that led to a sharp distribution of stimulus-triggered spikes for a similar neuronal network model [61]. This is in line with our results that suggest that these differences are a consequence of the qualitatively different distributions of stimulus-triggered spikes for vibrotactile burst stimuli [26] and for electrical single-pulse stimuli [61].

More studies are needed to understand how different stimulus shapes can be employed to modulate synaptic connections using brain stimulation. In the context of DBS, most research has been devoted to tuning the waveform of DBS pulses for improving the efficiency in initiating neuronal responses [100–103], in entraining neuronal spiking [104], and for improving acute therapeutic effects [105, 106]. One study that, indirectly, analyzed how different stimulus patterns modified cortico-subthalamic synapses was performed by Yamawaki et al. [11]. There, in dopamine-intact conditions, high frequency stimulation hardly affected synaptic weights, whereas low-frequency bursts caused LTP of cortico-subthalamic synapses. Our results suggest that the waveform of DBS stimuli as well as the numbers of spikes per burst and the intraburst frequency during DBS may have a strong impact on synaptic connections and, consequently, potential long-lasting aftereffects, e.g. during CRS of the STN [20–23].

### PMCS-induced decoupling of neuronal populations

Recent studies suggested decoupling stimulation in order to induce long-lasting desynchronization [60–62]. This is important in the context of brain disorders characterized by abnormal neuronal synchrony, such as PD [107].

We analyzed the capability of PMCS with different types of stimuli to decouple neuronal networks. To this end, we investigated which PMCS parameters led to a reduction of the mean synaptic weight during PMCS and how robust this decoupling was with respect to changes of the stimulation parameters, e.g., the stimulus shape, the PMCS pattern, and the stimulation frequency (Figs 6–8).

Decoupling during PMCS with short electrical single-pulse stimuli was robust with respect to variations of the PMCS pattern, characterized by the phase lags between stimulus trains delivered to different neuronal subpopulations (Fig 6). The fastest decoupling was observed when stimuli were simultaneously delivered to multiple neuronal subpopulations. In this case, PMCS utilized “decoupling by synchrony”, which was previously presented in Ref. [29]. Decoupling by synchrony occurs in networks with canonical STDP and longer axonal delays than dendritic delays during sharp collective neuronal spiking events [29, 30]. Based on the observation of a corresponding “coupling by synchrony” effect in networks with longer dendritic than axonal delays by Morrison et al. [28], we would expect that PMCS with simultaneous stimulus delivery to multiple subpopulations would strengthen synapses in such networks.

When burst stimuli were employed, simultaneous stimulus delivery to multiple neuronal subpopulations led to even faster weight changes than for single-pulse stimuli. However, it depended on the intraburst frequency whether PMCS led to a weakening or a strengthening of synapses. PMCS with high intraburst frequencies increased the mean synaptic weight whereas low intraburst frequencies led to a reduction of the mean synaptic weight for a wide range of phase lags between stimulus trains (Fig 7). Overall the weight dynamics induced by PMCS with burst stimuli was more sensitive to the phase lags between stimulus trains than the weight dynamics induced by PMCS with single-pulse stimuli.

The fastest decoupling was observed when stimuli were simultaneously delivered to all subpopulations (periodic stimulation) and burst stimuli with low intraburst frequency were used. For sufficiently low stimulation frequencies, such a PMCS pattern is comparable to theta burst stimulation. A recent study in PD patients studied therapeutic effects of STN theta burst DBS [108]. There, 5 Hz burst stimulation (100 ms stimulation ON followed by 100 ms stimulation OFF) was delivered to PD patients. The authors varied the intraburst frequency and observed pronounced acute effects on rigidity, tremor, and akinesia. While rigidity improved for low and high intraburst frequencies (50 Hz and 100 Hz, respectively), akinesia responded better to low, whereas tremor responded better to high intraburst frequencies. This provides evidence that the intraburst frequency has a strong impact on therapeutic effects. The authors also tested for long-lasting aftereffects that outlasted stimulation; however, they did not observe significant aftereffects. They discussed that this was due to a too short stimulation duration of about 20–30 min used in the experiments, which was substantially shorter than the 2 h duration used in the proof-of-concept study for CRS in PD patients [23]. In other studies, theta burst stimulation was delivered using repetitive TMS [70]. The authors observed long-lasting aftereffects on the amplitude of motor-evoked potentials and reaction time that depended on the burst duration (corresponding to the number of TMS pulses per burst). Long-lasting effects of periodic burst stimulation were also observed during DBS of the globus pallidus in 6-OHDA mice, an animal model for PD [109]. The burst stimulation was able to restore movement, whereas continuous HF DBS did not have such aftereffects. Showing that the stimulus pattern has a strong impact on therapeutic effects. A previous study by Mastro et al. [110] provided evidence that stimulation may have to induce neuron population-specific responses in order to induce long-lasting effects. Variations of the intraburst frequency of delivered burst stimuli led to modulations of these neuronal responses, suggesting an impact of the intraburst frequency on long-lasting effects of stimulation. However, it was not studied whether the observed long-lasting effects resulted from stimulation-induced synaptic reshaping.

Together, these studies provide evidence that periodic burst stimulation may indeed be a promising candidate for inducing long-lasting effects in neuronal networks. While our results point out the importance of the intraburst spike pattern for harnessing STDP, the presented experimental evidence also suggests that it may be important for recruiting neuronal target populations and inducing neuronal responses that are beneficial for long-lasting effects.

### PMCS compared to CRS RVS

The presented PMCS may be considered as a generalization of CRS with fixed sequence [17]. CRS with fixed sequence was used in earlier studies, originally, in networks without plasticity. There, CRS was administered with fixed sequence to induce cluster states and acute desynchronization [17, 19]. Later, CRS RVS was delivered in plastic neuronal networks to avoid the formation of sequence related clusters, which might occur during CRS with fixed sequence [18]. CRS RVS was also successfully administered in clinical and preclinical studies to induce long-lasting therapeutic effects in PD patients [23, 26] and related animal models [20], respectively. In Zeitler et al. CRS with slowly varying sequence was introduced [65]. During CRS with slowly varying sequence, the sequence of stimulus deliveries to the individual neuronal subpopulations is shuffled every *n* cycles. Thus, the variation of *n* allows for studying the transition between CRS RVS (*n* = 1) and CRS with fixed sequence (*n* → ∞).

During our detailed analysis of the decoupling aspects of PMCS, we found that CRS with a fixed sequence (corresponding to PMCS with phase lags Δ*α*_*k*_ = 1/*M*) leads to a complete decoupling of the LIF network for intermediate stimulation frequencies when single-pulse or burst stimuli with a small number of pulses are used (Fig 8B” and 8C”). Remarkably, for the same stimuli, CRS RVS led to decoupling in an even broader frequency range (Fig 8B’’’ and 8C’’’). This provides evidence that random shuffling of the stimulation sequence is indeed advantageous for the frequency robustness of long-lasting effects.

Our results presented in Fig 8 also suggest that varying the size and number of separately stimulated subpopulations may substantially improve decoupling effects. The PMCS pattern in Fig 8A’ corresponds to a CRS pattern with fixed sequence where one subpopulation is twice as large as the other one. We found that such stimulation leads to robust decoupling in a large portion of the considered interval of stimulation frequencies for single-pulse stimuli and short burst stimuli with high intraburst frequencies, or a variety of burst stimuli with low intraburst frequencies (Fig 8B’ and 8C’). In contrast, if stimulation is delivered to three equally sized subpopulations, the frequency range with pronounced decoupling effects shrinks substantially (Fig 8B” and 8C”).

In terms of a controlled modulation of the synaptic network structure, previous theoretical studies found that CRS RVS causes different dynamics of intra- and inter-population synapses. However, all inter-population synapses showed on average the same dynamics [61]. In contrast, CRS with fixed sequence (Δ*α*_*k*_ = 1/*M*) leads to different phase lags between separately stimulated subpopulations and potentially allows for inducing a larger variety of network structures.

### Delivery of PMCS patterns using brain stimulation

Our theoretical and computational results provide evidence, that PMCS might be suitable for inducing changes of the synaptic network structure in plastic neuronal networks. This may lead to novel therapeutic stimulation techniques that specifically counteract pathological network connectivity.

PMCS requires multiple stimulation sites that allow for the separate stimulation of different neuronal subpopulations. The dynamics of synapses interconnecting neurons within individual subpopulations are strongly affected by the shape of delivered stimuli. In contrast, phase lags between stimulus trains delivered to different neuronal subpopulations can be tuned to modulate the strengths of plastic synapses interconnecting these subpopulations.

Recently, extensive research has been devoted to multisite DBS electrodes, which possess a large number of stimulation contacts [111, 112]. Using directional steering, these electrodes allow for targeting individual neuronal subpopulations more precisely than conventional electrodes [111]. These electrodes may allow for implementing the PMCS approach in target brain areas for HF DBS in PD and other brain disorders. Preclinical and clinical studies delivering CRS through multisite DBS electrodes reported long-lasting therapeutic effects [20–23]. Long-lasting symptom relief in PD patients was also achieved using non-invasive vibrotactile stimulation [25, 26, 94]. There, different fingers receive vibratory burst stimuli at different times. We hope to motivate further research on delivering PMCS through DBS electrodes or non-invasively using vibrotactile stimulation.

To effectively apply PMCS, it is important to identify synaptic target populations for PMCS-induced up- or down-regulation. In the context of multisite DBS of basal ganglia nuclei, experimental evidence from animal models of PD suggests that dopamine-depletion leads to extensive synaptic reformations in the basal ganglia network [9]. Especially synaptic connections formed with STN neurons experience plastic reshaping after dopamine depletion [9, 113, 114]. While changes of synaptic strengths in response to electrical stimulation have been observed for different synaptic populations [10, 11, 14], more experimental studies that explore the dependence of these changes on the time lag between postsynaptic and presynaptic stimulation [14] would support hypotheses on efficient PMCS patterns targeting the individual synaptic populations. Note, in order to apply the PMCS approach, an exact knowledge of the STDP function is not required. In our manuscript, we showed instead that the outcome of PMCS can be predicted based on estimates of the mean rate of synaptic weight change as a function of the phase lag between stimulus deliveries to the postsynaptic and presynaptic neuronal subpopulation (Fig 3). These estimates may be obtained by delivering phase-shifted periodic stimulus trains to two subpopulations and measuring the resulting synaptic weight change. We hope that our manuscript motivates experimentalists to consider such experiments in future studies on STN and GPi stimulation. Long-lasting effects of current multisite stimulation techniques [20–23, 109, 110] might result from inducing plasticity of some or all of these synaptic connections suggesting a variety of synaptic target populations for a possible PMCS approach.

In future studies, we aim at exploring the effect of different DBS stimulation patterns on the synaptic weight dynamics in a more detailed model of the basal ganglia. In this model, we also aim at exploring PMCS approaches in which different nuclei are considered as different subpopulations to counteract synaptic reorganization of inter-nucleus connections in response to the degeneration of dopaminergic neurons, observed in animal models of PD [113, 115]. In this context it would be of interest to incorporate the somatotopic organization of the basal ganglia nuclei [116] and compare PMCS approaches in which stimuli are delivered to neuronal subpopulations in different nuclei that represent similar body parts or to subpopulations that represent different body parts. Such a study may provide testable hypotheses on suitable neuronal target populations.

In future projects, we also anticipate exploring the PMCS approach in more detailed computational models of other neurological disorders such as epilepsy.

## Conclusion

Harnessing brain stimulation-induced synaptic plasticity may provide novel treatments for several brain disorders. We presented PMCS, a multisite stimulation technique that allows for inducing a wide range of desired network structures in plastic neuronal networks by delivering phase-shifted stimuli to different neuronal subpopulations. In our computational and theoretical study, PMCS was tuned to down-regulate certain types of synaptic connections while up-regulating others, suggesting that PMCS could be used to specifically counteract pathological synaptic connectivity as, for instance, observed in animal models of PD. Our detailed analysis showed that PMCS-induced synaptic reshaping strongly depended on the shape of employed stimuli, indicating that the choice of employed stimuli might strongly affect long-lasting effects of various types of brain stimulation. We further presented a combined computational and theoretical approach that provides a framework on how to predict PMCS-induced network structures given network characteristics such as the STDP function. We hope that our work inspires future preclinical and clinical studies on PMCS and on the effect of stimulus parameters on stimulation-induced long-lasting therapeutic effects in patients suffering from neurological disorders that are associated with abnormal synaptic connectivity patterns.

## Methods

### Neuronal network model

We performed simulations of a network of *N* = 10^3^ excitatory LIF neurons with STDP that was originally presented in Ref. [60]. The dynamics of the *i*th neuron’s subthreshold membrane potential *V*_*i*_(*t*) was given by

$$\begin{array}{ccc} C_{i}\frac{dV_{i}}{dt} & = & g_{\text{leak}}\left(V_{\text{rest}}-V_{i}\right)+g_{\text{syn},i}\left(t\right)\left(V_{\text{syn}}-V_{i}\right)+I_{\text{stim}}\left(t\right)+I_{\text{noise},i}\left(t\right). \end{array}$$ (5)

*C*_*i*_ is the membrane capacitance. The terms on the right-hand side represent the leak current with leakage conductance *g*_leak_ and resting potential *V*_rest_; the excitatory synaptic input with time-dependent synaptic conductance *g*_syn,*i*_(*t*) and reversal potential *V*_syn_; the stimulation current *I*_stim_(*t*); and the noisy input current *I*_noise,*i*_(*t*). A spike was generated whenever *V*_*i*_(*t*) crossed the threshold potential *V*_th,*i*_(*t*),

$$\begin{array}{ccc} \tau_{\text{th}}\frac{dV_{\text{th},i}}{dt} & = & -(V_{\text{th},i}-V_{\text{th,rest}}). \end{array}$$ (6)

Then, *V*_*i*_(*t*) was set to *V*_spike_ for a duration of *τ*_spike_. Afterwards, *V*_*i*_(*t*) and *V*_th,*i*_(*t*) were reset: *V*_th,*i*_(*t*)→*V*_th,spike_ and *V*_*i*_(*t*)→*V*_reset_.

The synaptic conductances, *g*_syn,*i*_(*t*), obeyed the dynamics

$$\begin{array}{c} \tau_{\text{syn}}\frac{dg_{\text{syn},i}}{dt}=-g_{\text{syn},i}+\kappa \frac{\tau_{\text{syn}}}{N}\sum_{j\in G_{i}}w_{j\to i}\left(t\right)\sum_{l^{j}}\delta \left(t-t_{l^{j}}^{j}-t_{\text{a}}\right). \end{array}$$ (7)

*τ*_syn_ is the synaptic timescale. $t_{l^{j}}^{j}$ is the timing of the *l*^*j*^th spike of the presynaptic neuron *j* and *t*_a_ the synaptic axonal delay. We restricted our computational analysis to axonal delays. However, in our theoretical analysis, we also considered dendritic delays. The outer sum in Eq 7 runs over all presynaptic neurons *j* ∈ *G*_*i*_ connected to neuron *i*. *κ* is the maximal coupling strength and *w*_*j*→*i*_(*t*) the weight of the synapse connecting neurons *j* and *i*.

To model input from other neuronal populations, we fed independent Poisson input with firing rate *f*_noise_ into the neurons through excitatory synapses. The resulting input currents, *I*_noise,*i*_(*t*), were given by

$$\begin{array}{c} I_{\text{noise},i}\left(t\right)=g_{\text{noise,i}}\left(t\right)\left(V_{\text{syn}}-V_{i}\right). \end{array}$$ (8)

Here, the noise conductances, *g*_noise,i_(*t*), resulted from

$$\begin{array}{c} \tau_{\text{syn}}\frac{dg_{\text{noise},i}}{dt}=-g_{\text{noise,i}}+\kappa_{\text{noise}}\tau_{\text{syn}}\sum_{k_{i}}\delta \left(t_{k_{i}}-t\right). \end{array}$$ (9)

*κ*_noise_ is the noise intensity and $t_{k_{i}}$ the *k*_*i*_th spike time of the Poisson spike train fed into neuron *i*.

All parameter values were chosen according to Ref. [60]: *g*_leak_ = 0.02 mS/cm^2^, *V*_rest_ = −38 mV, *V*_reset_ = −67 mV, *V*_th,spike_ = 0 mV, *V*_th,rest_ = −40 mV, *τ*_th_ = 5 ms, *V*_syn_ = 0 mV, *τ*_syn_ = 1 ms, *t*_d_ = 3 ms, *κ* = 8 mS/cm^2^, *κ*_noise_ = 0.026 mS/cm^2^, and *f*_noise_ = 20 Hz. The membrane capacitances, *C*_*i*_, were distributed according to a Gaussian distribution with mean *μ*_C_ = 3 *μ*F/cm^2^ and standard deviation *σ*_C_ = 0.05*μ*_C_. The frequency and range of the resulting membrane potential oscillations matched recordings of oscillatory neurons in the rat STN [66], a major target region for DBS in PD [90].

Throughout the present paper, we considered a homogeneous network in which the probability for a synaptic connection from any neuron *i* to any neuron *j* ≠ *i* was fixed to 7%. A similar network model was considered in a previous study on vibrotactile fingertip CRS in PD [26].

### Periodic multichannel stimulation

In order to manipulate network connectivity, we delivered PMCS. During PMCS, phase-shifted periodic stimulus trains are delivered to *M* neuronal subpopulations (see schematic in Fig 1A). Individual neuronal subpopulations were obtained by dividing the total neuronal population into *M* equally sized subpopulations.

Each subpopuplation received stimuli periodically at frequency *f*. We will refer to a time period of duration 1/*f* during which each subpopulation receives one stimulus as a “PMCS cycle”. Stimulus onsets may occur at arbitrary, but fixed phases during PMCS cycles. We denoted the constant phase of the stimulus onset in subpopulation *k* by *α*_*k*_, *k* = 1, …, *M*. Thus, all possible PMCS patterns could be parameterized with the *M* − 1 phase lags Δ*α*_*k*_ = *α*_*k*+1_ − *α*_*k*_, *k* = 1, 2, …, *M* − 1, and the onset phase of the stimulus delivered to the first subpopulation, *α*_1_. We set *α*_1_ = 0 throughout the present paper.

We studied PMCS for electrical single-pulse and burst stimuli. Our electrical stimulus model was similar to the one used in previous computational studies, e.g., [56, 60–62], and described direct electrical stimulation of the neuronal soma, for instance during DBS.

Individual stimuli were charge-balanced and consisted of an excitatory and an inhibitory rectangular pulse with durations *ν*_e_ = 0.4*d*_*e*_ ms and *ν*_i_ = 0.8*d*_*e*_ ms, respectively. We considered asymmetric stimuli, *ν*_e_ ≠ *ν*_i_, as such pulses were considered in preclincal and clinical studies on CRS [20, 21, 23]. Charge-balanced pulses are typically used during DBS to avoid tissue damage [117]. The excitatory rectangular pulse had the amplitude $A_{\text{e}}=A_{\text{stim}}\mu /\nu_{\text{e}}$ and the inhibitory one the amplitude $A_{\text{i}}=-A_{\text{stim}}\mu /\nu_{\text{i}}$. Here, *μ* = (*V*_th,spike_ − *V*_reset_)/〈*C*_*i*_〉 and *A*_stim_ is the dimensionless stimulation strength. (*V*_th,spike_ − *V*_reset_) is the voltage distance between the maximum spiking threshold *V*_th,spike_ and the reset potential *V*_reset_. 〈*C*_*i*_〉 is the mean membrane capacitance. An electrical single-pulse stimulus is depicted in Fig 2D.

During burst stimuli, individual pulses occurred at a fixed intraburst frequency *f*_intra_. An electrical burst stimulus consisting of five pulses is depicted in Fig 2H.

### Spike-timing dependent plasticity

The dynamics of the synaptic weights, *w*_*i*→*j*_(*t*), was determined by STDP. We considered a nearest-neighbor STDP scheme in which weight updates were performed whenever either a postsynaptic or a presynaptic spike arrived at a synapse [84]. Similar schemes were used in previous computational studies on long-lasting effects of CRS [56–58, 61]. Corresponding weight updates, *w*_*i*→*j*_ → *w*_*i*→*j*_ + *W*((*t*_*j*_ + *t*_d_) − (*t*_*i*_ + *t*_a_)), were given by the STDP function [60, 67]

$$\begin{array}{c} W\left(\Delta t\right)=\eta \{\begin{array}{cc} \;e^{-\left|\Delta t\right|/\tau_{+}}, & \;\Delta t>0\\ 0, & \;\Delta t=0\\ -\frac{\beta }{\tau_{R}}\;e^{-\left|\Delta t\right|/\tau_{-}}, & \;\Delta t<0 \end{array}\;\;. \end{array}$$ (10)

Here, Δ*t* = (*t*_*j*_ + *t*_d_) − (*t*_*i*_ + *t*_a_) is the time lag between the arrival time of the backpropagating postsynaptic action potential (spike) at the synapse, *t*_*j*_ + *t*_d_, and the latest presynaptic spike arrival time, *t*_*i*_ + *t*_a_ (if the update was triggered by the arrival of a backpropagating postsynaptic spike at the synapse), or the time lag between the current presynaptic spike arrival time, *t*_*i*_ + *t*_a_, and the latest postsynaptic spike arrival time, *t*_*j*_ + *t*_d_ (if the update was triggered by the arrival of a presynaptic action potential at the synapse) [44]. Here, *t*_d_ and *t*_a_ are the dendritic and the axonal delays, respectively. In our simulations, we considered only an axonal delay of *t*_a_ = 3 ms and set the dendritic delay to zero. Our theoretical analysis was done for both axonal and dendritic delays.

*η* = 0.02 scaled the weight update per spike, *τ*_R_ = 4 yielded an asymmetry in the STDP decay times *τ*_+_ = 10 ms and *τ*_−_ = *τ*_+_*τ*_R_. The parameter *β* = 1.4 set the ratio of overall LTD, i.e. the integral of the STDP function over time lags that led to negative weight updates, to overall LTP, i.e. the integral of the STDP function over time lags that led to postive weight updates. These parameters led to bistability between a strongly connected state with synchronized neuronal activity and a weakly connected state with asynchronous neuronal activity [26].

Initially, synaptic weights were distributed according to a bimodal distribution *w*_*i*→*j*_ ∈ {0, 1} with mean weight 〈*w*(*t* = 0)〉 = 0.5. We found that this led on average to a faster relaxation towards a stationary state than a Gaussian or uniform distribution of the initial synaptic weights. Note that our result did not depend on the initial distribution of the synaptic weights as we waited until the system approached a stationary state before stimulation was delivered. In more detail, to prepare networks in the stable synchronized state, we performed simulations for a total of 3000 sec using the Euler method with integration time step *h* = 0.1 ms. Then, stimulation was switched on.

### Theoretical description of PMCS-induced synaptic weight dynamics

Following Ref. [27], we considered the dynamics of the weight *w*_*i*→*j*_(*t*) of a synapse connecting the presynaptic neuron *i* and the postsynaptic neuron *j*. *w*_*i*→*j*_(*t*) changed due to the weight updates that are given by the STDP function, *W*(Δ*t*), Eq 10. Thus, the synaptic weight dynamics was determined by the statistics of time lags, Δ*t*, between postsynaptic and presynaptic spike arrival times at the synapse [27, 118, 119].

We were interested in the mean rate of weight change, $J_{ij}\left(t,T\right)$, during a time interval [*t*, *t* + *T*],

$$\begin{array}{c} J_{ij}\left(t,T\right)=\frac{1}{T}\sum_{t_{i},t_{j}\in \text{pairs}}W(\left(t_{j}+t_{\text{d}}\right)-\left(t_{i}+t_{\text{a}}\right)). \end{array}$$ (11)

The sum runs over all pairs of presynaptic spike times, *t*_*i*_, and postsynaptic spike times, *t*_*j*_, that contributed to weight updates in this time interval. Here, *t*_a_ is the axonal and *t*_d_ the dendritic delay [44].

To calculate the expected weight change during ongoing stimulation, we followed the approach of Ref. [60] and calculated the expectation value $\langle J_{ij}\left(t,T\right)\rangle$, which is obtained by averaging over different realizations of stimulus-triggered spikes. To this end, we assumed a stationary dynamics and long time intervals, *T* ≫ 1/*f*. Here, *f* is the stimulation frequency. Furthermore, *T* is assumed to be long compared to interspike intervals and STDP is assumed to be slow compared to the other time scales. In simulations of the LIF model, on average about 3.5 collective spiking events per second occurred in the synchronized state. Under these assumptions, $\langle J_{ij}\left(t,T\right)\rangle$ becomes independent of the starting point, *t*, and the length, *T*, of the time interval and we can approximate $\langle J_{ij}\left(t,T\right)\rangle$ by its limit for long time intervals $\langle J_{ij}\left(t,T\right)\rangle \to \langle J_{ij}^{\infty }\rangle$.

To obtain the statistics of time lags between postsynaptic and presynaptic spike arrivals at the synapse, we followed the approach of Ref. [60] and assumed stimulation-controlled spiking in which neuronal spiking is controlled by the stimulus pattern. However, in contrast to Ref. [60], we allowed for complex spiking responses to individual stimuli. These may include bursts. In accordance with Ref. [60], we assumed that the distribution of neuronal spike times during a PMCS cycle only depended on the difference between spike times and the onset time of the latest stimulus. This applies if each stimulus leads to a phase reset of the stimulated neuronal subpopulation and if synaptic interaction is weak, i.e., synaptic input is unlikely to trigger neuronal spikes during a PMCS cycle. For periodic stimulation patterns, such as PMCS, this also applies if stimulation leads to an entrainment of the neuronal spike or burst rhythm.

We characterized neuronal spiking responses by the conditional probability densities of interspike intervals, λ_*l*_(*ϵ*_*l*−1_|*x*_*l*−1_). Here, λ_*l*_(*ϵ*_*l*−1_|*x*_*l*−1_) is the conditional probability density of the (*l* − 1)th interspike interval, *ϵ*_*l*−1_, after stimulus onset given that the (*l* − 1)th spike occurred at time *x*_*l*−1_ after stimulus onset. λ_1_(*ϵ*_0_) ≔ λ_1_(*ϵ*_0_|0) is the conditional probability of the “zeroth” interspike interval, referring to the time interval between stimulus onset (*t* = 0) and the first spike, given that the “zeroth” spike, referring to the stimulus onset, occurred at time zero. $x_{l}=\sum_{k=0}^{l-1}\epsilon_{k}$ is the time lag between the *l*th spike and the stimulus onset time. Here, we only considered interspike intervals between spikes that occured before the next stimulus onset. Thus, individual λ_*l*_(*ϵ*_*l*−1_|*x*_*l*−1_) were not necessarily normalized as the spike at time *x*_*l*−1_ might have been the last spike before the next stimulus onset. By assuming that the conditional probability densities of interspike intervals only depended on the timing of spikes relative to the stimulus onset and not on the entire sequence of interspike intervals, we neglected correlations in the sequence of interspike intervals. Such correlation patterns may, for instance, arise from slow intrinsic dynamics such as adaptation currents, see for instance Ref. [120]. Furthermore, we assumed a homogeneous network in which spike times of each neuron were described by the same conditional probability densities.

For a given sequence of λ_*l*_(*ϵ*_*l*−1_|*x*_*l*−1_), we calculated the probability densities Λ_*l*_(*x*_*l*_) of the *l*th spike times *x*_*l*_ after stimulation onset as

$$\begin{array}{c} \Lambda_{l}\left(x_{l}\right)=\int_{0}^{1/f}d\epsilon_{l-1}\ldots \int_{0}^{1/f}d\epsilon_{1}\int_{0}^{1/f}d\epsilon_{0}\delta (x-\sum_{k=0}^{l-1}\epsilon_{k})\lambda_{l}(\epsilon_{l-1}|\sum_{m=0}^{l-2}\epsilon_{m})\ldots \lambda_{2}\left(\epsilon_{1}|\epsilon_{0}\right)\lambda_{1}(\epsilon_{0}). \end{array}$$ (12)

**Fig 14:**
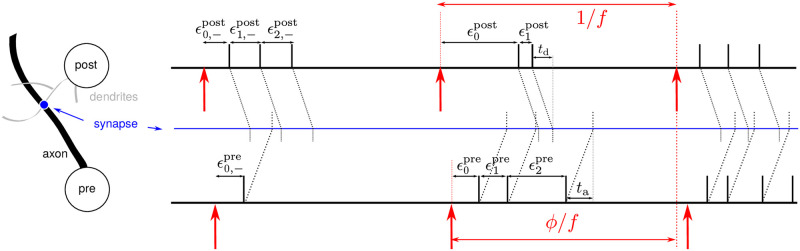
Schematics of the derivation of the estimated mean rate of weight change. During PMCS, the postsynaptic neuron and the presynaptic neuron (left) received stimuli at frequency *f*. Stimulus deliveries (red arrows) were phase shifted by a phase lag *ϕ* ∈ [0, 1]. Each stimulus could trigger several spikes (vertical bars). Presynaptic spikes traveled along the axon and arrived with a time delay *t*_a_ at the synapse (blue). Backpropagating postsynaptic spikes traveled along the dendrites and arrived with a delay time *t*_d_ at the synapse. Interspike intervals *ϵ* are marked by horizontal arrows. Indices count interspike intervals during respective PMCS cycles. Dashes refer to interspike intervals from the previous PMCS cycle. Suffixes ‘post’ and ‘pre’ indicate interspike intervals of the postsynaptic or presynaptic neurons, respectively.

In order to calculate $\langle J_{ij}^{\infty }\rangle$, we considered the statistics of negative time lags corresponding to weight updates that were triggered by presynaptic spike arrivals, and the statistics of positive time lags corresponding to weight updates that were triggered by postsynaptic spike arrivals at the synapses (Fig 14). We calculated the expectation value of the total weight update that resulted from positive time lags during one PMCS cycle, *W*^+^, and that of the total weight update resulting from negative time lags, *W*^−^. To obtain the total positive weight update, we considered the statistics of arrival times, *x*^*j*^ + *t*_d_, of the *m*th postsynaptic spike at the synapse, relative to the presynaptic arrival times, *x*^*i*^ + *t*_a_. We found

$$\begin{array}{ccc} & & W_{m}^{+}\left(\phi ,f,\xi \right)\approx \sum_{k=-\infty }^{\infty }[\\ + & & \sum_{n=1}^{\infty }\int_{0}^{\infty }dx^{i}\Lambda_{n}\left(x^{i}\right)\int_{0}^{\infty }d\epsilon^{i}\lambda_{n+1}\left(\epsilon^{i}|x^{i}\right)\int_{\frac{k-\phi }{f}+x^{i}+\xi }^{\frac{k-\phi }{f}+x^{i}+\epsilon^{i}+\xi }dx^{j}\Lambda_{m}\left(x^{j}\right)W(x^{j}-(\frac{k-\phi }{f}+x^{i})-\xi )\\ + & & \int_{0}^{\infty }dx^{i}\Lambda_{\text{last}}\left(x^{i}\right)\int_{0}^{\infty }d\epsilon^{i}\lambda_{1}\left(\epsilon^{i}\right)\int_{\frac{k-\phi }{f}+x^{i}+\xi }^{\frac{k+1-\phi }{f}+\epsilon^{i}+\xi }dx^{j}\;\;\Lambda_{m}\left(x^{j}\right)\;\;W(x^{j}-(\frac{k-\phi }{f}+x^{i})-\xi )], \end{array}$$ (13)

with *ξ* = *t*_a_ − *t*_d_. *ϕ* is the phase lag between stimulus trains delivered to the postsynaptic and presynaptic neurons (Fig 14). The second row sums over the cases where the *m*th postsynaptic spike arrived between the *n*th and the (*n* + 1)th presynaptic spikes of the presynaptic spiking/bursting response triggered by a stimulus arriving *k* PMCS cycles later than the stimulus that triggered the postsynaptic spike (a negative *k* refers to an earlier stimulus). The third row accounts for cases where the postsynaptic spike arrives between the last spike of that PMCS cycle and the first spike of the next PMCS cycle.

Accordingly, we find

$$\begin{array}{ccc} & & W_{m}^{-}\left(\phi ,f,\xi \right)\approx \sum_{k=-\infty }^{\infty }[\\ & + & \sum_{n=1}^{\infty }\int_{0}^{\infty }dx^{j}\Lambda_{n}\left(x^{j}\right)\int_{0}^{\infty }d\epsilon^{j}\lambda_{n+1}\left(\epsilon^{j}|x^{j}\right)\int_{\frac{k+\phi }{f}+x^{j}-\xi }^{\frac{k+\phi }{f}+x^{j}+\epsilon^{j}-\xi }dx^{i}\Lambda_{m}\left(x^{i}\right)W(\frac{k+\phi }{f}+x^{j}-x^{i}-\xi )\\ & + & \int_{0}^{\infty }dx^{j}\Lambda_{\text{last}}\left(x^{j}\right)\int_{0}^{\infty }d\epsilon^{j}\lambda_{1}\left(\epsilon^{j}\right)\int_{\frac{k+\phi }{f}+x^{j}-\xi }^{\frac{k+1+\phi }{f}+\epsilon^{j}-\xi }dx^{i}\Lambda_{m}\left(x^{i}\right)W(\frac{k+\phi }{f}+x^{j}-x^{i}-\xi )]. \end{array}$$ (14)

Here, Λ_last_(*x*) is the probability density that the last spike during a PMCS cycle occurred at time *x*. It follows from the sequence of Λ_*k*_(*x*) of the *k*th spike times *x* and the conditional probability densities λ_*k*+1_(*ϵ*|*x*) as

$$\begin{array}{c} \Lambda_{\text{last}}\left(x\right)=\sum_{k=1}^{\infty }[1-\int_{0}^{\infty }d\epsilon \;\lambda_{k+1}\left(\epsilon |x\right)]\Lambda_{k}\left(x\right). \end{array}$$ (15)

We calculated the probability *P*(*k*) to have exactly *k* spikes during a PMCS cycle as

$$\begin{array}{c} P\left(k\right)=\int_{0}^{\infty }dx\;[1-\int_{0}^{\infty }d\epsilon \lambda_{k+1}\left(\epsilon |x\right)]\Lambda_{k}\left(x\right). \end{array}$$ (16)

Then, we approximated the total weight updates resulting from positive and negative time lags as

$$\begin{array}{c} W^{+}\left(\phi ,f,\xi \right)\approx \sum_{k=1}^{\infty }P\left(k\right)\sum_{m=1}^{k}W_{m}^{+}\left(\phi ,f,\xi \right),\\ W^{-}\left(\phi ,f,\xi \right)\approx \sum_{k=1}^{\infty }P\left(k\right)\sum_{m=1}^{k}W_{m}^{-}\left(\phi ,f,\xi \right). \end{array}$$ (17)

Finally, the total weight update per PMCS cycle is approximately given by

$$\begin{array}{c} W\left(\phi ,f,\xi \right)\approx W^{+}\left(\phi ,f,\xi \right)+W^{-}\left(\phi ,f,\xi \right). \end{array}$$ (18)

Together with the PMCS frequency this yields the estimated mean rate of weight change

$$\begin{array}{c} \langle J^{\infty }\left(\phi ,f,\xi \right)\rangle \approx fW\left(\phi ,f,\xi \right). \end{array}$$ (19)

Eq 19 was used throughout the paper to calculate the expected rate of weight change $\langle J^{\infty }\left(\phi ,f,\xi \right)\rangle$.

To calculate the mean rate of weight change, Eq 19, we evaluated Eqs 13 and 14 numerically. For *ϵ*, *x*^*i*^, and *x*^*j*^, we introduced bins of size 0.2 ms and discretized the time interval *t* ∈ [0, 1/*f*). The infinite sums in Eqs 13 and 14 were truncated after *k* = −5 and *k* = 5 during the numerical evaluation. In Eq 17, the sum over *k* was truncated for the maximum number of spikes per PMCS cycle observed during the simulation from which the estimated conditional probability densities of interspike intervals were obtained (see below).

### Estimation of conditional probability densities of interspike intervals using simulation data

We estimated the conditional probability densities of interspike intervals, λ_*k*_(*ϵ*_*k*−1_|*x*_*k*−1_), for the LIF network with STDP. To this end, we delivered periodic stimulation with frequency *f* for 100 seconds to 333 (≈ *N*/*M*) neurons and recorded the stimulated neurons’ spike timings relative to stimulus onset times. Exemplary raster plots for the LIF network for this setup are shown in Fig 2A and 2E. An estimate of the average number of stimulus-triggered spikes was obtained by calculating the mean number of spikes per PMCS cycle (see Fig 2B for single-pulse and Fig 2F for burst stimuli, respectively).

Results for the cumulative distribution function $F_{1}\left(t\right)=\int_{0}^{t}du\;\Lambda_{1}\left(u\right)$ are shown in Fig 2C for single-pulse and in Fig 2G for burst stimuli. These estimates were used to calculate the mean rate of weight change from Eq 19. The results are shown in Fig 3. The latter was used to obtain theoretical estimates of the mean synaptic weight from Eq 4 (Figs 5, 6, 7 and 8) and estimates of the block structure from Eq 1 (Figs 4, 5 and 8–13).

### Estimation of mean rate of weight change from simulations

In Fig 3, we show data points for the estimated mean rate of weight change, $J_{\text{est}}$, of synapses between subpopulation one and two obtained from simulations of the LIF network model. These results were obtained by recording the mean weight of these synapses, 〈*w*_1→2_(*t*)〉, every 50 ms. Prior to stimulation this mean weight had a value of about 〈*w*_1→2_(*t* < *t*_0_)〉 ≈ *w*_0_ ≈ 0.38. Here, *w*_0_ is the mean weight of all synapses. To estimate the mean rate of weight change, we then only considered data points with 0.1 < 〈*w*_1→2_(*t* < *t*_0_)〉 < 0.9 and applied python’s numpy polyfit function to get an estimate of the slope, $J_{\text{est}}^{†}$, using a linear fit (degree one polynomial) of either the increase or the decay of 〈*w*_1→2_(*t*)〉 during stimulation (*t*_0_ > *t*). From these slopes, we obtain $J_{\text{est}}$ from $J_{\text{est}}=J_{\text{est}}^{†}/\left(1-\langle w_{1\to 2}\left(t_{0}\right)\rangle \right)$, if $J_{\text{est}}^{†}>0$, and $J_{\text{est}}=J_{\text{est}}^{†}/\langle w_{1\to 2}\left(t_{0}\right)\rangle$, if $J_{\text{est}}^{†}\leq 0$.
